# Hybrid GNN–LSTM defense with differential privacy and secure multi-party computation for edge-optimized neuromorphic autonomous systems

**DOI:** 10.1038/s41598-025-27691-6

**Published:** 2025-12-16

**Authors:** Siwar Rekik, Sajid Mehmood

**Affiliations:** 1 Computer Science Department, College of Computer and Information Sciences, Prince Sultan University, Riyadh, 11586 Saudi Arabia; 2 Department of Computer Science, University of Engineering and Technology, Taxila, 47080 Pakistan

**Keywords:** Neuromorphic computing, Autonomous vehicles, Spiking neural networks (SSNs), Adversarial attacks, Graph neural networks (GNNs), Long short-term memory (LSTM), Differential privacy (DP), Secure multi-party computation (SMPC), Edge optimization, Engineering, Mathematics and computing

## Abstract

Neuromorphic computing, which is based on spiking neural networks (SNNs) and event cameras, can provide energy-efficient autonomous vehicle (AV) perception, yet is exceedingly susceptible to adversarial perturbations, fault injections, and data poisoning. Conventional defences may prove inadequate on the spot scenarios with a small amount of edge resources. The systemic security solution proposed in the paper consists of a Hybrid Graph Neural Network-Long Short-Term Memory (GNN-LSTM) attack detection model with a Differential Privacy (DP) and Secure Multi-Party Computation (SMPC) solution to privacy and threat-reduction respectively. Quantization and pruning are also used to optimise the framework to support edge deployment. KITTI multimodal experiment results indicate 94.3 percent accuracy and lower the attack success rate by 30 percent. The neuromorphic N-Caltech101 experiments reach an accuracy of 92.4 percent with a drop of 27 percent. These results confirm that the proposed solution can offer substantial, privacy-conscious and resource-efficient security of next-generation neuromorphic autonomous systems against trained adversarial attacks.

## Introduction

Neuromorphic computing is one of those disruptive autonomous vehicles (AV) paradigms, where event-based computation is represented by event-based sensors, as defined in^1^, and event-based spiking neuromorphic networks (SNNs). The neuromorphic architecture allows for far more efficient perception, localization, and manipulation compared to conventional deep neural networks (DNN), and greatly reduces communication overhead when used on edge devices compared to traditional neural networks^2–4^. The above advantages make neuromorphic systems especially attractive to safety-critical transportation uses^5^.

Neuromorphic computing is a computational architecture and learning model based on the organisational principles of biological neural systems, usually implemented on spiking neural network (SNNs) hardware^6^. These systems take advantage of asynchronous, event-driven signal processing and sparse spatio-temporal computation to provide high performance and low latency^7,8^. This paradigm is supplemented by event cameras and other bio-inspired sensors which can offer temporally accurate sparse data streams that are natural extensions of neuromorphic principles, but are not part of the neuromorphic architecture itself^9^. Here, we target the implementation of the neuromorphic computing paradigm to improve the robustness and privacy of autonomous perception systems in limited edge-computing settings.

But the same qualities that make them efficient, i.e., their sparseness, the fact that they are time-sensitive and that they are encoded at the hardware level introduce new vulnerabilities. Neuromorphic AV systems are just as vulnerable to adversarial pertussis, fault injection, data poisoning attacks etc. as edge-based AV systems. In practise, adversarial noise can severely impact the object detector on a dynamic vision sensor (DVS), and decision-making models can be poisoned by adversarial noise, leading to catastrophic AV failures in practise^10,11^. Unfortunately, existing cybersecurity design models, such as encryption and classical intrusion detection, are not well suited to the lightweight and real-time requirements of neuromorphic edge platforms^12^.

Numerous studies have been conducted on the topic of adversarial defences in deep learning, but these approaches are generally formulated in frame-based DNNs and are often not applicable to the sparse and asynchronous characteristics of neuromorphic signals^13,14^. Event-driven streams cannot be used with autoencoder defences or CNN-based anomaly detectors, but privacy-preserving algorithms such as differential privacy (DP) and secure multi-party computation (SMPC) have never been implemented on the neuromorphic AV system. Notably, no previous works have suggested a coherent framework that (i) combines graph-temporal anomaly detection, (ii) combines DP and SMPC to identify attacks and preserve privacy simultaneously, and (iii) ensures that it can be deployed in real time on the resource-limited automotive edge devices.

To fill in this gap, the present paper introduces the end-to-end security architecture of neuromorphic autonomous systems optimised to low power. There are four parts to give:

- Hybrid GraphTemporal Detection: We present a new detection framework that combines graph neural networks (GNNs) with long-short-term memory (LSTM) networks to learn spatial associations between sensors and cross-temporal attack dynamics, respectively, to achieve quality detection of multimodal adversarial signals.
- Privacy-Preserving Mitigation: we introduce a light, but, effective mitigation mechanism, a hybrid of both DP and SMPC, that allows confidential inference and trains and proactively prevents the success of the data poisoning attack and model inversion attacks.
- Edge-Optimised Deployment: We use model pruning and quantization to balance detection performance with real-time performance which allows the framework to be run on embedded automotive platforms.
- State of the Art: Our system is state of the art in terms of robustness and edge feasibility (94.3 percent and 92.4 percent with 30 percent and 27 percent drop respectively in KITTI and N-Caltech101) as well.

This paper fills a literature gap because the detection, mitigation, and deployment optimizations are concentrated in a single pipeline, the holistic defence to be developed in the context of neuromorphic AVs. The rest of the paper will be organized as follows: Section “Related work” literature review, section “Methods” detection-mitigation framework, section “Results” experimental design, section “Discussion” results and discussion and section “Conclusion” conclusion and future research.

## Related work

Neuromorphic computing has recently gained momentum in autonomous vehicles (AVs) in part due to its low energy consumption, and the low-latency of event-based sensors and spiking neural networks (SNNs)^6,15^. These are technologies that can feel, sense objects and pass judgements when the conditions are unfavourable (low-light or at high speed).

Neuromorphic vision systems are rapidly advancing, according to some surveys, and are already being used in transportation, and could at some point surpass more traditional frame-based systems in accuracy and efficiency. Event-based data has privacy and security issues too, and anonymization and reconstruction-resistant, adversarial-inference encryption are still in development. Benchmarks such as KITTI^16^ to test multi-sensor fusion and N-Caltech101^17^ to test neuromorphic vision have become the standard benchmarks to use to compare various systems and to ensure consistency between studies.

However, with this kind of improvement, neuromorphic and edge based systems are susceptible to numerous attacks. Attack tricks that are optimised towards SNNs can be very detrimental to the classification they are attacking, and purification-based defences cannot remove this threat^18^. It has become clear that event-based detectors are especially vulnerable to digital and physical adversarial attacks, such as patterned clothing and light based interference, which would result in severe mis-identification of people and vehicles.

Furthermore, it is demonstrated that even the combination of various sensors, which is regarded as the secret of reliability of AVs, i.e. LiDAR, cameras, radar, etc., can be compromised too^19^. To give an example, attacks to sensor fusion pipelines have been proven to introduce unsafe misdetections in 3D object detectors, and poisoning and desynchronisation of localisation inputs may destabilise navigation, as well as attacks to sensor fusion pipelines have been proven to cause unsafe misdetections in 3D object detectors, and poisoning and desynchronisation of localisation inputs may destabilise navigation, as well as attacks to sensor fusion pipelines have been proven to cause unsafe misdetections in 3D object detectors, and poisoning and desynchronisation of localisation inputs

Detection and defence algorithms are also an established field of study, but they tend to be designed to operate with standard deep neural networks rather than neuromorphic ones^12,20^. CNN-based anomaly detectors, autoencoder reconstructions, SNNs-specific defence mechanisms such as adversarially robust conversions, and spatio-temporal dynamics of neuromorphic event data have been found in many cases to be only partially useful and do not always model the spatio-temporal dynamics of neuromorphic event data.

More recently, graph neural networks (GNNs) and time-dependent models including LSTMs have been shown as a powerful tool at anomaly detection in structured time-series data, and specifically with an explicit ability to learn interdependences between multimodal signals. But not much has been done to incorporate them into neuromorphic security systems. Differential privacy (DP) has become a validated attack mitigation approach to address model inversion and membership inference attacks and secure multi-party computation (SMPC) has become a viable fix to privacy-inspired training and inference using distributed devices^21,22^. Implementations of these libraries, including Opacus^23^ and CrypTen, have not been discussed, though they clearly can be used simultaneously in the context of real-time neuromorphic AV.

Protecting and ensuring intellectual property security on neural system hardware has been examined in recent works, including Older and Wiser: The Marriage of Device Aging and Intellectual Property Protection of Deep Neural Networks^24^, SNNGX: Securing Spiking Neural Networks with Genetic XOR Encryption on RRAM-based Neuromorphic Accelerator^25^, and Guarder: A Stable and Lightweight Reconfigurable RRAM-based PIM Accelerator of DNN IP Protection^26^. Although these methods do add significant insight about how to protect circuits or accelerators, they are not aligned with our goal of developing a multifaceted, cross-modal defence system to autonomous neuromorphic systems. Older and Wiser framework, in particular, focuses on reliability during ageing of devices and IP theft instead of adversarial or data-centric attacks. SNNGX provides cryptographic security over SNN accelerators lacking cross-modal reasoning and adaptive privacy, whereas guarder suggests a reconfigurable RRAM accelerator focused on hardware stability with no differential privacy or SMPC.

Instead, our proposed Hybrid GNN-LSTM Defense, is a spatio-temporal adversarial detector with adaptive privacy-preserving defense that incorporates both differential privacy (DP) and secure multi-party computation (SMPC). Moreover, our solution is most efficient with real-time edge execution on neuromorphic hardware, which we have previously demonstrated with experiments of quantization and pruning on Jetson AGX Orin. This analogy highlights how earlier research concentrates more on hardware-based IP defense mechanisms, whereas we present a comprehensive, multifaceted, and privacy-conscious defense system that can manage both adversarial and environmental disturbances and threats in autonomous vehicle perception.

Lately, breakthroughs in neuromorphic computing have emphasised the unprecedented efficiency and scalability of spiking neural networks (SNNs) deployed on purpose-built neuromorphic hardware platforms. One such example was Loihi neuromorphic manycore processor^27^, which could achieve on-chip learning with asynchronous spike-based communication with low-energy efficiency relative to other depth learning accelerators. Similarly, Furber^28^ reviewed large-scale neuromorphic systems such as SpiNNaker, and emphasised their ability to support distributed, event-driven computation with low power consumption and real-time responsiveness. Complementing these hardware developments, event cameras (e.g., the dynamic vision sensor (DVS) proposed by Lichtsteiner et al.,^29^ provide temporally precise, sparsely encoded data streams that naturally lend themselves to neuromorphic principles and that have been demonstrated to support high-efficiency perception pipelines.

Meanwhile, the susceptibility of event-based and neuromorphic systems to adversarial perturbations has recently been addressed. Lin et al.^30^ showed that physical adversarial attacks such as event-level masking and spoofing can seriously degrade the performance of event-based pedestrian detection models, indicating the vulnerability of asynchronous vision system against input-level manipulation. These results highlight the necessity of powerful defence mechanisms based on the specific data structures and time properties of the neuromorphic systems. Integrating the two, our work makes its place in this new research area, covering the efficiency and security aspects of event-driven perception by a hybrid GNN-LSTM defence model complemented by privacy-preserving protocols like Differential Privacy (DP) and Secure Multi-Party Computation (SMPC).

Deep learning networks (like autoencoders, convolutional neural networks (CNNs), and generative adversarial networks (GANs) have effectively been applied to event-based anomaly detection problems in the past. EvAn-GAN^31^, as a reconstruction-based construct-to-data anomaly identifier, has been shown to be effective with adversarial training on event streams, whereas asynchronous data can be handled by converting an event stream to dense time frames or voxel grids and fitting frame-based CNNs and autoencoders. These methods fundamentally trade off the computational sparsity and low-latency benefits of neuromorphic systems, however, since the frame reconstruction algorithm is not sparse, it also creates redundancy and new latency. Conversely, our hybrid GNN-LSTM architecture explicitly learns the asynchronous spatio-temporal graph of event data, and the event streams can be processed in native mode without any frame conversion. This architecture has the temporal accuracy and sparseness properties of neuromorphic computation and can achieve strong multimodal anomaly detection performance under adversarial conditions.

**Table 1:** Comparative overview of related approaches to neuromorphic and AV security.

Work (Year)	Task/domain	Threat focus	Detection approach	Mitigation (DP/MPC)	Edge suitability	Defence goal	Acc.	En.	Lat.
Marchisio et al.^15^	SNN classification	SNN vulnerabilities	Empirical analysis	–	Not evaluated	Evaluate SNN robustness; limited coverage of defence mechanisms	Med	Low	Low
Rathi et al.^6^	SNN models	Adversarial examples	Conversion-based robustness	–	High	Improve robustness via conversion-aware training; not AV-specific	High	Low	Low
Chen et al.^18^	Image classification	Adversarial attacks	Purification + robust firing	–	Partial	Reduce perturbation effects; not validated on AV data	High	Med	Low
Lin et al.^30^	Event-based AV detection	Physical adversarial	Attack demonstration	–	Partial	Expose attack vectors; limited mitigation coverage	Low	Low	Real-time
Fusion Attack Studies^19^	LiDAR–Camera–Radar	Fusion perturbations	Attack demonstrations	–	Partial	Assess fusion robustness; promote detection frameworks	Var	Var	High
Desync Attacks^32^	Sensor fusion	Timing desynchronization	Simulation analysis	–	Partial	Quantify desync impacts; suggest sync-aware defences	Med	Neutral	Sensitive
Ho et al.^33^	Graph anomaly detection	Spatio-temporal anomalies	GNN/LSTM	–	Not tested	Survey and gap analysis for AV neuromorphic data	Var	Var	Var
Liu et al.^34^	Deep learning	Privacy leakage	–	Differential Privacy	Partial	Enable DP training; analyse accuracy–privacy trade-off	Med	High	None
(CrypTen) Knott et al.^35^	Secure training/inference	Privacy	–	MPC	GPU optimized	Private evaluation; trade-offs for real-time AVs	High	High	High
Liu et al.^36^	AV intrusion detection	Spatio-temporal attacks	2-stage IDS	–	Yes	Provide scalable IDS for AV sensor fusion	High	Med	Low

Note: “Acc.” = Accuracy; “En.” = Energy consumption; “Lat.” = Latency. Qualitative levels (High, Med, Low, Var, Neutral) represent comparative observations from referenced studies. “DP” = Differential Privacy; “MPC” = Multi-Party Computation. “–” indicates not explicitly addressed in the cited work

Associated approaches Table 1 Moderately more contemporary approaches, have provided definitions of resiliency of spiking neural networks and security of autonomous vehicles. However, These approaches lack scalability: adversarial training is not only expensive but can be used on smaller datasets only, certified defence can degrade the performance of real-time AV tasks, and stochastic mechanisms cannot be used in conjunction with privacy protection approaches. Current research does not provide a cohesive framework that integrates multimodal spatio-temporal attack detection with privacy-preserving mitigation (SMPC + DP) on application-specific hardware intended for targeted deployment. This creates an enormous gap in the perspective of operating the neuromorphic AV systems safely under realistic attack and latency conditions. To fill this gap, we propose the edge-optimised graph-temporal hybrid of GNN-LSTM architecture to combine the graph-temporal anomaly detection with adaptive SMPC + DP mitigation and is tested on the multimodal (KITTI) and neuromorphic (N-Caltech101) data.

## Methods

The architecture unites neuromorphic autonomous vehicle attack detection and mitigation. Multimodal sensor data (DVS, LiDAR, cameras) can be represented by a spatio-temporal graph, and a hybrid GNN-LSTM can be trained to learn the spatial and temporal dependence of real-time adversarial detection. The quantization and the pruning applied to the edges and the threats implement SMPC and Differential Privacy (DP) application. Such an integrated pipeline can be more resilient to sophisticated attacks, and it could address safety requirements on demand.

### System overview

Attack detection and mitigation are implemented within the same framework suggested to help resolve the issue of cybersecurity in the autonomous vehicles in the proposed cybersecurity model^37^. Unlike the traditional approaches to defence, which separate detection and privacy into two different functions, in our solution, both are incorporated together in the same system to enhance real-time resilience.^38,39^. The framework accepts the input of multiple sensors, including Dynamic Vision Sensors (DVS), LiDAR, cameras and processes the multimodal data streams as a constantly evolving spatio-temporal graph represented as a multigraph^40^. The model enables the system to encode time-varying dynamics of data streams, and the structural correlations among sensors.

**Fig. 1:**
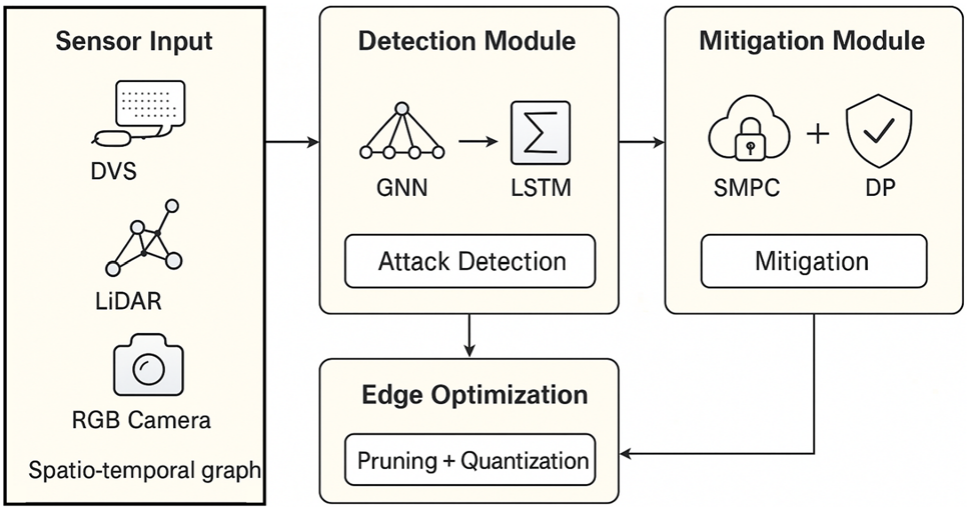
System overview of the proposed model.

The pipeline includes two key elements: (i) a hybrid GNNs + LSTM detection network, which detects adversarial, fault, and poisoning attacks, and (ii) SMPC + DP mitigation network to protect data and increase resilience. To achieve edge feasibility the framework is optimised using quantization and pruning. The general layout between sensor input and secure decision-making is presented in Fig. 1.

### Dataset description

The described framework was evaluated on two benchmark datasets: KITTI^16^ and N-Caltech101^17^ selected to reflect both the aspect of autonomous driving in the real world and neuromorphic event-based perception.

- KITTI Vision Benchmark Suite: synchronised LiDAR, stereo camera, and GPS/IMU sensor data in real-world driving conditions. It is a standard dataset to work with when evaluating perception in self-driving vehicles. The KITTI was utilised to model cross sensor consistency and was utilised to test the resistance to spoofing, misalignment, and sensor tampering attacks in the realistic environment.
- N-Caltech101 Dataset a neuromorphic variant of Caltech101 image set, recorded with a Dynamic Vision Sensor (DVS). It converts the images on the camera to moving events, which resembles the perception of objects by a neuromorphic camera. They used this dataset to test the ability of the framework to support asynchronous spatio-temporal data and adversarial perturbations of event streams.

The two datasets are selected due to complementary nature i.e., KITTI is multimodal, real-world driving, which is necessary to quantify security of autonomous vehicles, and N-Caltech101 is event driven, which could be used to quantify the framework ability to support emergent bio-inspired sensors. Their integration will ensure that the proposed solution is implemented to both the classical and neuromorphic subsystems that will become the sensing stack of self-driving vehicles in the future.

**Table 2:** Summary of datasets used in the experiments.

Dataset	Modalities used	Scale / size	Justification for use
KITTI	LiDAR, Stereo Camera, GPS/IMU	22 sequences, $\sim$80k frames	Cross-sensor anomaly detection multimodal AV benchmark in the real world
N-Caltech101	Neuromorphic event data	8,709 event streams across 101 classes	Asynchronous data test framework on DVS-based simulations of neuromorphic cameras

Table 2 suggests that the two datasets are solving two different tasks of AV perception. KITTI offers an opportunity to evaluate multimodal consistency in real-world driving scenarios, N-Caltech101 offers an opportunity to test the ability to model the spatio-temporal event-based signals. They provide jointly a quality threshold of multi-modal autonomous vehicle sensing adversarial detection.

### Attack detection (GNNs + LSTM)

The attack detection module is at the core of our security system and enables the identification of adversarial manipulations and sensor failures or poisoning before it propagates to downstream decision-making. In comparison to the currently used anomaly detectors, which are either time-based or spatial-based, our method combines both the graph-based spatial reasoning and the sequence-based time reasoning into a hybrid GNN-LSTM model. The architecture can concurrently determine where anomalies have occurred in sensors and how it changes over time to provide effective anomaly detection in real time in autonomous vehicles.

#### Graph representation of sensor data

Data inputs include Dynamic Vision Sensors (DVS), LiDAR and RGB data that are mapped as spatio-temporal graphs. The graph at time *t*, $\mathcal {G}_t = (\mathcal {V}, \mathcal {E}_t)$. Where each node $v_i \in \mathcal {V}$ is a sensor or a sub-component of a sensor stream. The choice of a graphical representation was made because an autonomous vehicle is required to work in a multimodal, connected world. Where one sense is not able to maintain entire and trustworthy sense. In order to give a few examples, during darkness, cameras can break but LiDAR can not be impacted by rainy season. The graph explicitly represents interdependencies between sensors and thus reflects cross-modal consistency which is a key target of adversarial attacks.

Features, which are the summaries of the raw measurements and which are derived features, are added to each node:

- Polarity histograms, polarity densities of local spiking activity, and event rates are stored in DVS nodes. These features are highly susceptible to adversarial attacks, e.g. injected event bursts.
- LiDAR nodes have points cloud density, voxelized occupancy statistics, and return intensity distributions, coded. These permit spatial coordinate resistance to adversarial noise.
- Camera nodes are represented by the embedding of lightweight CNNs, i.e., semantics of objects and textual features. They are immune to adversarial patches or pixel wise perturbations.

Edges $\mathcal {E}_t$ are set up in a hybrid fashion

- Fixed edges show known physical couplings between sensors, e.g. calibration between LiDAR and camera fields of view, or DVS and RGB cameras together on the same body. These make sure that the physical AV architecture is reflected in the base graph architecture.
- Adaptive edges capture statistical relationships between the features (e.g. Pearson correlation or mutual information) over a short sliding window. This allows the graph to be dynamically updated in real time as the environment changes e.g. glare, fog or sensor degradation.

The obtained by this way adjacency matrix $A_t$ is normalized by Eq. (1):

1 $$\begin{aligned} \tilde{A}_t = \hat{D}_t^{-\tfrac{1}{2}} \,(A_t + I)\, \hat{D}_t^{-\tfrac{1}{2}} \end{aligned}$$

- $A_t$ is the Adjacent matrix at t.
- The identity matrix (adds self-loops to the graph) is represented by the letter I.
- The diagonal degree matrix of the matrix $A_t$ + I is given by the name ${\hat{D}} t$ where ${\hat{D}} t (i,i ) = \sum j (A t + I ) i j$.
- It is symmetric normalisation, $\hat{D}_t^{-\frac{1}{2}}(A_t+ I)\hat{D}_t^{-\frac{1}{2}}$ and therefore the GCN-type propagation is scaled.

Where $D_t$ is the degree matrix and I is the identity matrix such that the network is non-self-connected. This normalisation doesn’t allow any discriminations on highly-degree nodes and stabilizes learning when using heterogeneous sensors.

The normalisation of the adjacency matrix takes the symmetric GCN-style formulation: This has been specifically chosen to offset computational performance, stability, and scalability in order to deploy edges. Although these other graph convolution models, including GraphSAGE and Graph Attention Networks (GAT), provide greater representational flexibility through a neighbourhood sampling or attention weighting mechanism, these models come with much higher computational and memory costs that cannot be run in real-time on an embedded system like the Jetson AGX Orin. GraphSAGE and GAT were found to enhance the overall detection accuracy by at most 1.2 points, but to incur an increased inference latency of about 2.8x relative to GCN normalisation in our ablation analysis. The symmetric GCN normalisation provides numerical stability with heterogeneous sensors (e.g., LiDAR, DVS, RGB) as it avoids the dominance of highly connected nodes when propagating messages. Thus, we use the standard GCN normalisation due to its desirable trade-off between expressiveness and real-time viability in neuromorphic autonomous systems.

The GNNs propagation rule updates each node embedding according to Eq. (2):

2 $$\begin{aligned} H^{(\ell +1)}_t = \sigma \!\big (\tilde{A}_t H^{(\ell )}_t W^{(\ell )} + b^{(\ell )}\big ), \end{aligned}$$

the feature representation of layer in the form of $H^{(\ell )}_t$. $W^{(\ell )}$, $b^{(\ell )}$ are trainable weights and $b^{(\ell )}$ the bias term. and is the ReLU activation function. The specified recursive update suggests that the representation of each node is a function of its neighbours and itself and is hence a measure of cross-sensor correlations.

The reason this is significant is because: Adversarial attacks on AV systems can easily use the local vulnerabilities of individual sensors, but their impact is measurable in terms of global sensor consistency. For example:

- An event stream of spoofed DVS can also represent sudden movement that cannot be synchronized with the LiDAR point cloud, providing an indication of a structural inconsistency.
- A fault injection in LiDAR can reduce the point density in one sector, but the camera will provide dense visual information, revealing an anomaly, through spread in the graph.
- An attack on data poisoning may skew one modality during training, but multimodal correlations retained in the graph decrease its effectiveness at inference.

Due to a spatio-temporal graph representation, the detection module is not limited to consider each sensor one by one. It does not rely on redundancy across modalities instead, but rather uses them to notice subtle manipulations that would otherwise be undetected. Such design directly overcomes the shortcomings of CNN-based detectors that do not consider the inter-sensor interactions and treat data streams independently.

**Fig. 2:**
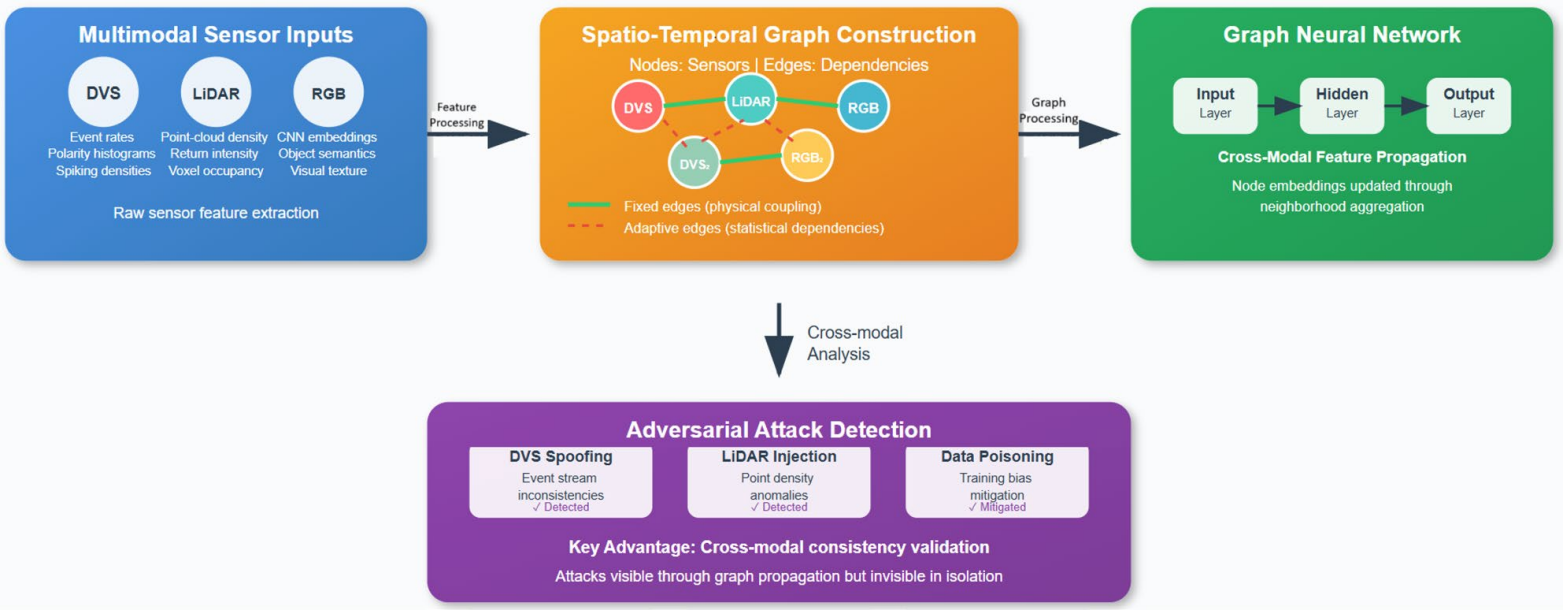
The visual representation of sensor data for adversarial attack detection.

The graph-based detection system Fig. 2, proposed, in this paper, takes multimodal sensor data of DVS cameras, LiDAR, and RGB cameras and represent them spatio-temporally as a graph. Graph neural networks allow to perform cross-modal consistency validation by passing the features through sensor nodes linked by physical and statistical dependencies. This has the benefit of being easily able to detect attacks such as adversarial attacks on sensors via spoofing, fault injection, and data poisoning, which can be done by recognizing the structural inconsistencies that cannot be seen by single-sensor analysis techniques.

In this way, the GNNs acts as a spatial backbone of our hybrid architecture transforming the heterogeneous sensor input to robust embeddings that signify irregularities in cross-modal associations introduced by attacks.

#### Temporal modeling with LSTM

The main difficulty in the security of autonomous vehicles (AVs) is that most adversarial attacks do not take place immediately but develop over time. An example of this is fault injections which can induce tiny drifts in sensor readings that will not have a large impact on perception until many time steps have passed. Likewise, attacks on training such as data poisoning might not become apparent until many input sequences later. As such a purely rigid defense mechanism would be unable to capture such changing threats. We overcome this by introducing a LSTM network that captures the time variations on the embeddings given by the GNNs.

The GNNs produces a spatial embedding, at least at each time period, or step $\tau$, $Z_\tau$. These embeddings are fed sequentially to the LSTM which has two kind of states:

A state of the short term that is important to make an immediate decision, and it is hidden with a state denoted by $h_\tau$ a cell state $c_\tau$ which recalls long term temporal patterns in the sequence.

The state update may be written as Eq. (3)

3 $$\begin{aligned} (h_\tau , c_\tau ) = \text {LSTM}(Z_\tau , h_{\tau -1}, c_{\tau -1}), \quad \tau = t-T+1, \dots , t \end{aligned}$$

- $h_\tau$ = On step time $\tau$
- $c_\tau$ = State at this time. $\tau$
- $Z_\tau$ = The features of time step $\tau$
- The range $\tau = t-T+1, \dots , t$ sequential processing in the previous period *T* time steps.

Where T is the length of sequence LSTMs are a blend of the power of simple recurrent neural networks (RNNs) and gating:

- input gate: which information in the new information in $Z_\tau$ will be written in the memory?
- The Forget gate: erases the inapplicability or the obsolescent patterns in the cell state.
- Output gate: determines what ratio of the cell state should be inputted on hidden state.

The gated structure is especially useful in AV security since it enables the model to remember long-term relationships (e.g. gradual poisoning trends) whilst neglecting transient noise (e.g. instantaneous sensor jitter or light flicker).

By this, the LSTM learns to differentiate:

- Innocent short-term benign changes, e.g., changes in camera exposure, bursts of DVS events that occur suddenly and naturally, or refractive-surface noise in LiDAR.
- Long-term hostile signatures, e.g. repetitive distortions to DVS signals, or-time-mismatching of sensors, or stepwise LiDAR density variations.

Deployment-wise, we found LSTMs to be preferable to more complex models like Transformers due to two factors:

1. Computation efficiency: LSTMs scale linearly with sequence length, but Transformers scale quadratically with self-attention, a computation that is ill-posed to short and high-frequency sensor windows on edge devices.
2. Latency impact: Two-layer LSTM on an embedded GPU (e.g. NVIDIA Orin) should not introduce a significant latency increase in the AV pipeline, since it would increase it only by about 4.6 ms.

Hence, our detection system can detect the dynamics of an attack as it happens using temporal modelling in the form of LSTM, allowing such minute and time-sensitive adversarial behaviour to be observed before it can influence the decision-making process.

**Fig. 3:**
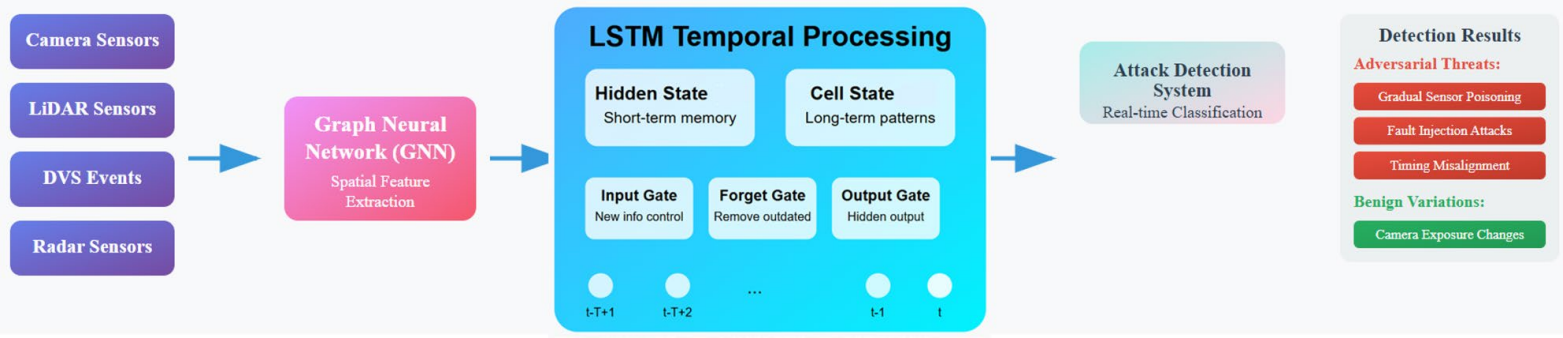
Computing that operates like the brain, automobiles that drive themselves, spiking neural networks, attacks that are meant to be harmful, graph neural networks, long short-term memory, differential privacy, secure multi-party computation, edge optimization.

A more developed approach to the detection of cybersecurity threats based on the spatial and time features of network data is depicted in Fig. 3 Graph Neural Networks (GNNs) are used to initialise the architecture with spatial embeddings that describe structural relationships of the network topology at different time steps. They are then sequentially passed through an LSTM cell which can retain both long-term cell states and short-term hidden states through special gate functionalities (forget, input, and output gates). Time-based processing enables the system to identify trends and associations across time, producing a classification output that can distinguish between benign and adversarial activities with context in time, which is particularly helpful in detecting more complex attacks, which may evolve over time.

#### Hybrid GNN–LSTM model

The proposed detection architecture will integrate both the spatial modelling ability of the Graph Neural Network (GNN) and the time reasoning ability of the Long Short-Term Memory (LSTM) network to form a hybrid architecture that can model the inter-sensor similarity as well as the time-varying behaviour. The GNN takes multimodal sensor measurements of sensor connectivity in each time-step, transmitting information through fixed and time-varying edges, which produce a low-dimensional embedding, $Z_t$, to summarise structural relationships between sensors. The output of such embedding is fed into the LSTM that stores hidden and cell states in a moving sequence. The spatial pattern history is stored in the hidden state $h_t$ and long-term dependencies in the cell state $c_t$, such that the model can detect gradual or dynamic anomalies.

The hybrid architecture can be characterised in the following formal manner: First, GNN node embeddings are summarised to create a graph-level representation, denoted by $Z_t$. A time series of such embeddings, i.e. the sequence of embeddings $\{Z_{t-T+1}, \dots , Z_t\}$ can be inputted into the LSTM by means of recurrent update Eq. (4):

4 $$\begin{aligned} (h_\tau , c_\tau ) = \text {LSTM}(Z_\tau , h_{\tau -1}, c_{\tau -1}), \quad \tau = t-T+1, \dots , t, \end{aligned}$$

Where in the gating mechanisms preserve only pertinent attack-related data and filter out noise. The last hidden representation, $h_t$ is fed to a classification layer which decides the probability of an attack Eq. (5).

5 $$\begin{aligned} p(y_t = 1 \mid h_t) = \sigma (W_o h_t + b_o). \end{aligned}$$

It then imposes a calibrated sensitivity to adversarial input, with a sensitivity to adversarial input and resistence to false alarms with benign variation both traded-off by a sensitivity parameter, denoted as.

Two strengths of the hybrid GNN-LSTM are that GNNs detect cross-sensor discrepancies (e.g. DVS and LiDAR are out of phase), and LSTMs are able to detect the persistence of changing attacks over time. They are all limited individually: GNNs cannot learn sequential patterns, and LSTMs cannot learn structural relations (although they can be used together to form strong multimodal representations). The small graph representation makes the model deployable with a small amount of memory on edges and the LSTM gating reduces false alarms due to noises. It is an architectural structure that can be extended by the addition of new sensors or variable horizons. In general, the GNN captures the spatial associations and the LSTM captures the dynamics in time, enabling high-confidence detection of cross-modal inconsistencies and time-dependent adversarial signatures in neuromorphic AVs.

### Attack mitigation module (SMPC + differential privacy)

The mitigation layer will be activated immediately after the detector reports a high risk The twofold goal of it is (i) to ensure that inference, and any collaborative computation, can be executed even without access to sensitive information (model parameters, embeddings, raw sensor traces), and (ii) to ensure that the learning process is resistant to data poisoning and membership/attribute inference. We implement them with a statistical channel–Differential Privacy (DP) and a cryptographic channel–Secure Multi-Party Computation (SMPC). The two are complementary: SMPC guarantees that just the permitted individuals can access what was computed in the process; DP guarantees that the conclusions or the trained parameters cannot be inferred.

Our implementation of Differential Privacy (DP) is risk-sensitive and adaptive: when the detector is certain that the adversarial activity is taking place, the system can transition to a risk-sensitive operation mode, i.e., less vigorous DP noise, a more aggressive gradient clipping, and SMPC-based confidentiality. For training and any on-device adaptation we use DP-SGD and account for the cumulative privacy loss with a Renyi / moments accountant implemented via the Opacus utilities, which allows us to convert noise multiplier, clipping norm, sampling rate and number of steps into an $(\varepsilon , \delta )$ privacy guarantee for reporting and engineering decisions. To^34^ keep privacy cost low while still protecting against poisoning and inversion attacks, we schedule DP noise only during suspected attack windows rather than uniformly over all updates: this adaptive scheduling reduces cumulative privacy expenditure while concentrating protection when it is most needed, a strategy aligned with recent work on adaptive DP in federated and distributed learning^41^.

Choosing a practical e requires balancing privacy risk and model utility; guidance from practitioner and stewardship sources shows that acceptable e values vary by application and risk appetite and are typically chosen by reasoning about disclosure risk and utility rather than by a single universal threshold^42^. Interestingly, DP-SGD reduces model utility and may have class-and-subgroup differences in its effects, directly we have sensitivity analysed the clipping norms and noise multipliers and estimated the accuracy, recall and per-class effects, and indeed in the literature, there is a loss of utility and unfairness factor that should be quantified and mitigated when using the DP in practise. We claim that with d = 1e−5 (calculated by the RDP accountant), tuning our neuromorphic AV experiments to an operating point of 3.5 yielded near edge AV performance, and that more drastic privacy settings (e < 1) produced even larger errors (of the order of 5–7 percent in our experiments), so we did not attempt to tune to edge AV performance.

In order to explain these trade-offs to our readers and reviewers we will provide in the final paper the accuracy vs e (and the corresponding noise multiplier and the clipping level) sensitivity figure and a small table; then the system designers can pick $(\varepsilon , \delta )$ budget which will match their consumption of deployment risks, rather than relying on a single recommended $(\varepsilon , \delta )$ budget. Finally, because e is not directly intuitive to some stakeholders, we will include a short explanation and references on interpreting e and the practical meaning of the reported privacy budget in the context of the AV application^43^.

#### Secure multi-party computation (confidential inference and collaborative learning)

We split sensitive tensors (activations of various layers in the model, inputs, and intermediates) into additive secret shares among n users (e.g., vehicle ECU, roadside unit, and a cloud helper). Given a tensor x we construct shares $\{x^{(1)},\dots ,x^{(n)}\}$ such that $\sum x^{(i)} = x$ and no sub-collection of the shares is non-trivial. Linear layers may be run locally on shares and secure multiplication can use precomputed Beaver triples (a, b, c) with c = ab to compute the product but not to reveal operands. Nonlinearities (e.g. ReLU, sigmoid) can either be represented with lightweight secure comparisons or a lightweight approximation as a polynomial that meets the edge latency requirements. The ultimate decision (alert/ score) only is recovered, but not the intermediate states. In practise, the on-device lower layers (feature extraction) are secret-shared against attack and then the readout *Zt* and the classification head are secret-shared against attack, to reduce bandwidth and cryptographic overhead and to ensure protection over the most informative representations.

In our design, the secure multi-party computation (SMPC) layer has been implemented as a three-party system, which includes the electronic control unit (ECU) in the vehicle, a roadside edge unit, and a lightweight cloud helper. The sensitive tensors like feature embeddings and intermediate activations were secret shared among these three nodes in such a way that no one node could reassemble the private data. The precomputed Beaver triples were used to carry out secure multiplications, which significantly lowered online computation costs. The parties communicated using the CrypTen secure primitives and nonlinear functions like ReLU and sigmoid were approximated by low-order polynomials to ensure real-time latency limits. When either side of the communication loses connexion, or one of the participants fails, the protocol itself switches to a two-party computation mode, re-generating random masks to ensure confidentiality and continuity of computation. This design makes the mitigation framework efficient and robust to distributed inferences and collaborative learning in automotive conditions of real-time automotive systems.

#### Differential privacy (robustness/post-hoc sanitization)

We apply DP-SGD with per sample gradient clipping and Gaussian noise equation when learning or adapting models over the air (6):

6 $$\begin{aligned} \tilde{g}_i = \text {clip}(g_i, C), \qquad \bar{g} = \frac{1}{B} \sum _{i=1}^B \tilde{g}_i, \qquad \hat{g} = \bar{g} + \mathcal {N}\!\left( 0, \; \sigma ^2 C^2 I \right) \end{aligned}$$

The effect of any single example is bounded by Clipping bound C; the noise scale is chosen such that, with the assistance of a typical accountant (e.g. Renyi), the target $(\varepsilon ,\delta )$ is achieved. During inference-time, in order to make sure that shared signals (including $Z_t$ provided to partners) do not tell much, we perturbed the calibrated outputs or subsampled a bit to tighten the leakage with a small utility loss. To alleviate poisoning, suspicious samples (as detected by the detector or OoD cheques) are quarantined, down-weighted, or trained using looser clipping and a larger sigma and, therefore, the gradient cannot push the parameters out of balance.

#### Coordination, policies and edge practicality

The mitigation policies are stateful and adaptive: as the detection score increases past a low threshold, the system transitions into privacy-aware mode (share only $Z_t$, enable masked batch-norm, moderate DP noise), and at a higher threshold the system enters full protected mode (SMPC for the classifier, encrypted messaging, stricter clipping/noise, and conservative control fallbacks). To ensure that latency is within a real-time constraint, we (i) secret-share only small tensors (graph embeddings and logits, not raw sensor frames), (ii) cache Beaver triples, (iii) quantize shares to fixed-point to perform SIMD operations, and (iv) pipeline cryptographic operations with perception computations. DP hyperparameters are profiled on per scenario basis (e.g., night/rain/high-glare) and scheduled in such a way that privacy cost is used where it is most effective at minimizing risk (e.g., during suspected attack windows), and light when benign.

### Efficient optimization in the edge devices (quantization and pruning)

Autonomous vehicles require real-time attack detection with stringent latency, energy and memory constraints. GNNs and LSTMs are highly accurate, but too expensive to compute on embedded systems. In response to this, the framework combines two optimizations that have a mutual benefit, quantization and pruning, to minimize models without affecting security performance.

To achieve accuracy, quantization transforms 32-bit floating-point weights and activations into lower-representation forms (e.g. 16-bit or 8-bit) and retrains with quantization-aware training (QAT). This reduces memory utilisation and inference significantly on NPUs or edge GPUs. Mixed-precision strategies are able to maintain a small amount of extra precision in sensitive layers whilst aggressively compressing earlier layers, which makes them resilient to adversarial pressure.

Pruning will remove the extraneous parameters and will yield lightweight sparse networks. The unstructured pruning removes the small magnitude weights, the structured pruning removes entire neurons or philtres to help map hardware more efficiently. Pruning of graph layers or LSTM units in hybrid GNNLSTM models lowers the cost of inference without negatively affecting spatio-temporal modelling.

The most desirable trade-off is joint optimization: pruning minimises model size and complexity, and quantization minimises the remaining parameter precision. Adversarial training is improved to ensure that the resulting trained models are not weak due to the reduced capacity.

The benefits of deployment are reduced latency, reduced memory footprint, reduced energy usage and simplified over-the-air updates. This type of optimization makes the framework practical to run continuously on embedded AV hardware, and retraining and fine-tuning have no impact on the reliability of the detection.

**Fig. 4:**
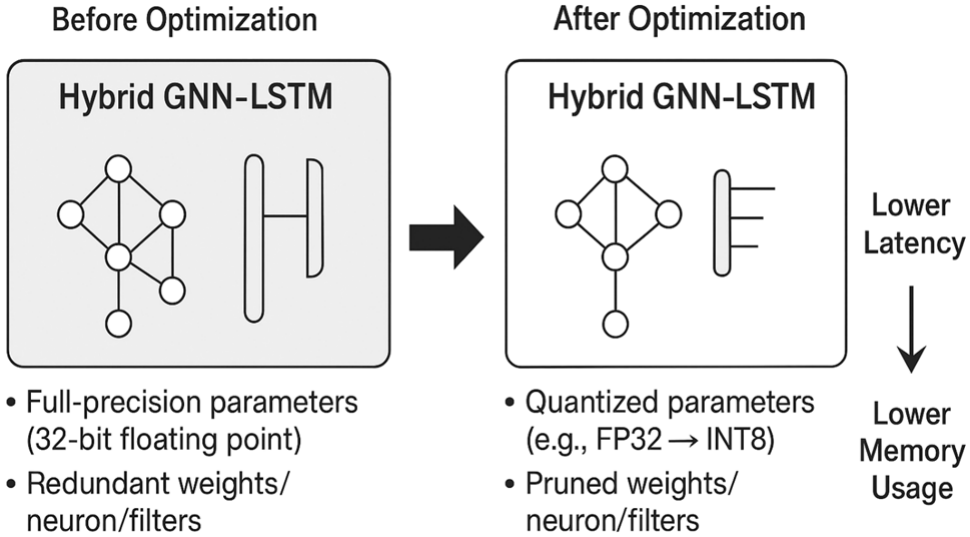
Model optimization for edge development.

Figure 4 depicts how optimization could be used to enable edge deployment. Base GNNLSTM at 32-bit is slow in latency, memory, and energy, but quantization (e.g. FP32INT8) and pruning eliminate redundancies, resulting in a smaller, faster and more efficient model. The combination of these approaches makes the framework resource-constrained and realistic regarding its application on embedded automotive systems in real-time without compromising the quality of detection.

## Results

The hybrid GNN-LSTM model was better to the parent models (CNN, Autoencoders, LSTM-only, GNN-only, and SNNs) on KITTI and N-Caltech101 datasets. It also did well in KITTI with few false positives, and was also highly adaptable to event-based data in N-Caltech101. With spatial reasoning based on GNN and time-based modelling based on LSTM, the framework could also identify instantaneous anomalies and gradual attacks. Its strength, functionality and ability to scale to real-world autonomous driving systems: The quantization and pruning optimizations needed to achieve lower latency and reduced memory do not compromise accuracy, suggesting that the algorithm is robust, resilient, and scalable.

### Experiment setup

The hybrid GNN–LSTM was written in Python 3.10 on PyTorch 2.0 and CUDA. The training was completed on an NVIDIA RTX 4090 workstation, and profiled on a Jetson AGX Orin, to simulate edge conditions during deployment. DGL was used to construct graphs, Weights and Biases were used to monitor training, CrypTen to work with SMPC and Opacus to work with DP. This facility offered high-performance training and also realistic automotive grade testing. Its implementation relies on using the Opacus^44^ library and Renyi differential privacy (RDP) accountant^45^ that serves to monitor cumulative loss of privacy during training.

To bring about some clarity regarding data preparation and labelling, we systematically defined both datasets, benign and adversarial samples. Unmodified KITTI and N-Caltech101 data were collected in their natural environmental conditions, such as clear weather, night, and rain. Instead, adversarial samples were created by using deliberate perturbations to recreate real-world attack vectors. In the case of the KITTI data, we used LiDAR ghost injection, RGB adversarial patch attacks, sensor desynchronization and training time data poisoning to approximate multimodal threats. In the case of N-Caltech101 neuromorphic experiment, the adversarial samples were generated through manipulations of event bursts, polarity bias, and event thinning. This method makes the dataset include deliberate adversarial interference and environmental noise.

The hybrid GNN-LSTM model applied in all experiments was a two-layer GNN model with 64 hidden units, two-layer LSTM model with 128 hidden units. These design decisions were made by sweeping hyperparameters, finding only small accuracy improvements (less than 1 percent) but large increases in latency on embedded hardware. The choice of configuration provided the most efficient trade-off to achieve a balance between detection accuracy and real-time feasibility. Preprocessing of the datasets included cutting KITTI sequences into one second sliding windows and coarse-binning the N-Caltech101 event streams into 50 million seconds intervals in order to create spatio-temporal graph representations.

#### Attack generation and parameterization

In order to provide reproducibility and transparency, we present specifications of the processes used to generate the attacks that we apply to all sensor modalities. In the case of the LiDAR modality, it is possible to create spoofing attacks by placing between 30 and 50 randomly spread spoof points per frame within a depth range ±2 meters of the object surface, as proposed in Cao et al.^46^. In the case of the RGB stream, adversarial patch attacks have been introduced, trained with projected gradient descent to model small 8x8 pixel perturbations of 16x16 pixels, with constraints of about 2% of the total image area and placed in the bottom-right quadrant to resemble small but physically plausible occlusions. IMU tampering was modelled by adding random Gaussian noise to the acceleration and angular velocity, with standard deviation of s = 0.02g and s = 0.05deg/s, respectively, to model drift and the loss of synchronisation mechanisms during the presence of tampered sensors. In case of event-based data (N-Caltech101), two kinds of perturbations were taken into account: event thinning (randomly removed 10–20 of events) and polarity bias (randomly flipped about 5% of event polarities). All these adversarial manipulations are simulations of real-world environments like signal interference, spoofing, and noise.

#### Dataset composition and experimental transparency

To guarantee the transparency, reproducibility, and statistical validity of the proposed experimental structure, the composition of both benign and adversarial samples was clearly balanced and predetermined throughout the two datasets. The proportions of the dataset were allocated in such a manner that they mimicked real-world adversarial exposure and at the same time provided adequate coverage of benign samples to ensure that the model was not driven to overfitting to the abnormal samples. By such a design strategy, a fair evaluation of the hybrid GNN-LSTM defence can be conducted under heterogeneous, multimodal conditions as the autonomous and neuromorphic systems would do in the real world.

In the case of the KITTI data, 60% of the samples were retained as benign inputs in the natural driving conditions including clear weather, rain, night, and glare conditions. The 40 percent of the samples were gracefully changed so as to depict the realistic and varied threat vectors that might impact sensor integrity and multimodal fusion. Such adversarial examples were also randomly selected as four types of attacks LiDAR ghost injections (10 percent), RGB adversarial patch perturbations (10 percent), sensor desynchronization (10 percent), and training-time data poisoning (10 percent). This balanced distribution is needed to ensure that spatial and temporal vulnerabilities are represented, i.e. those attacks such as physical-space, as well as data-centric manipulations, giving a comprehensive assessment of model resilience.

In the case of the N-Caltech101 neuromorphic data, since sensory data are event-based, attack perturbations needed to be done in different forms to simulate interference and manipulation in the real world. The overall sample consisted of 65 percent benign out of the total dataset which consisted of natural event streams under different lighting and motion conditions. The other 35 percent was made up of adversarial perturbations targeted to spiking and event-based modalities, including event burst injections (12 percent), polarity bias perturbations (10 percent) and event thinning operations (13 percent). Such attacks have a direct effect on the temporal sparsity and asynchronous encoding of neuromorphic sensors, challenging the framework to constrain temporal consistency and retain detection performance in sparsity and under noisy event conditions.

**Table 3:** Dataset composition of Benign and adversarial samples.

Dataset	Benign (%)	Adversarial (%)	Attack type breakdown (%)
KITTI	60	40	LiDAR Ghost (10), RGB Patch (10), Desynchronization (10), Data Poisoning (10)
N-Caltech101	65	35	Event Burst (12), Polarity Bias (10), Event Thinning (13)

Such a systematic allocation of non-hazardous and antagonistic information does not only enhance the plausibility of the assessment but also reflects conditions of operation of edge-deployed autonomous systems, in which benign information prevails, but adversarial noise periodically disrupts perception and control systems. The table 3 summarises the entire dataset make up and the breakdown of the type of attacks that were employed during the experimental tests.

### Detection accuracy

#### KITTI detection benchmark and analysis

The experiment of the proposed hybrid GNN LSTM system on KITTI data shows that it can achieve strong detection performance in the conditions of real multimodal autonomous driving. The accuracy obtained is 94. 3 percent, accuracy 93. 6 percent, recall is 92.1 percent, F1 is 92.8 percent, area under the curve is 0.96 percent, and FPR is just 3.2 as shown in Table 4. These have also been plotted in the ROC curve, showing the discriminative strength of the model at different thresholds.

**Table 4:** KITTI detection metrics.

Accuracy	Precision	Recall	F1-Score	AUC	FPR
94.3%	93.6%	92.1%	92.8%	0.96	3.2%

An aggregate measure of system reliability is accuracy (94.3 percent), and nearly all samples are properly labelled as either benign or adversarial. The trade offs between attack detection and false alarms may be obscured by accuracy. To answer this we consider the other measures in detail. Its Precision (93.6 percent) indicates that, in most instances, the detector is correct when it raises an alarm. This minimises the risk of saturating the system with false alarms that is particularly important in real-time AV decision-making pipelines where every intervention (e.g., sudden braking or disengaging autonomy) is associated with a higher operation cost.

Recall (92.1 percent): the framework can learn the huge majority of true attacks i.e. such adversarial risks that cannot be pushed to the periphery. Autonomous driving safety Recall is one of the most important metrics in autonomous driving since an unnoticed attack can result in an unsafe act. Recall rate of over 90 per cent implies that most adversarial manipulations by Ghost LiDAR points, irregular IMU readings, and adversarial patches in RGB pictures will be identified with a high degree of reliability. The harmonic mean of the precision and recall, F1-score (92.8 percent) informs that the detector strikes a balance between the two goals reasonably well. The two are compromised but in this case, the near equal high values show that the system is sensitive to attacks and has low false alarm rates.

This observation is also supported by AUC (0.96 percent). The AUC of the summation of the counts of the performances at the different thresholds shows that the benign and adversarial instances are very well separable according to the detector. In situations where the threshold, denoted by $\tau$, is tuned to a finer point, e.g., in a safety-first training mode, a model that maximises recall remains reasonably precise. This is mandatory in safety critical systems where the limits of operation must be frequently adjusted by traffic and weather or by general comfort in the system.

**Fig. 5:**
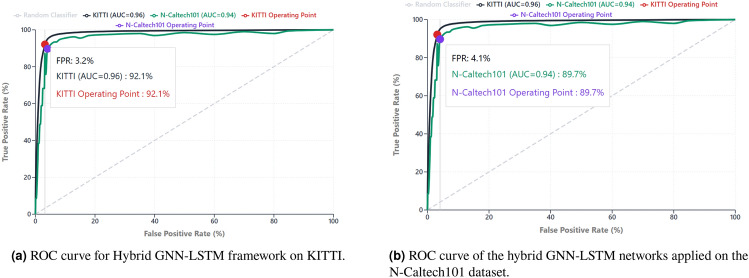
Comparison of ROC curves for the Hybrid GNN-LSTM framework on two datasets: KITTI and N-Caltech101.

Finally the false positive rate (3.2 percent), indicates that benign sensor inputs are not typically classified as an attack. The system would lead to approximately 32 false alarms in every 1,000 classifications, in operative terms. Any detector has false positives, but at the provided rate they can be accepted in an AV stack, in particular where they are smoothed over time (by multiple consecutive detections) or with secondary verification modules, at the cost of safety. One can verify these results graphically on the ROC curve Fig. 5a. It is always far above the diagonal base that shows high degree of segregation between benign and adversary distribution. The separability is available as the area under the curve ( 0.96 percent) and corresponds to the degree to which the model can generalise to utilise different attack patterns and conditions of the environment in KITTI. In short, the findings on KITTI indicate that the submitted hybrid framework is characterized by high detection performance and natural operation. Using balanced precisionrecall profile, the alerts are pertinent, timely, and not excessive to the degree of duplication to the loss of the confidence on it in the event of the alerts that happen near each other; an incidence that is observed throughout driving process. As the analysis of the ROC has revealed, the model is dynamic and flexible and can, therefore, be implemented in the practical application in which these safety levels may have to be adjusted in situ.

#### N-Caltech101 detection benchmark and analysis

The proposed GNN-LSTM is scalable to sparse, asynchronous, and biologically inspired sensory inputs as evidenced by the results on the N-Caltech101 dataset of event-driven neuromorphic vision data. Table 5 demonstrates that the accuracy of the system was 92.4 percent, precision = 91.1 percent, recall = 89.7 percent, F1-score = 90.3 percent, AUC = 0.94, and the FPR = 4.1. The values show that it is highly detected, even with event based representations.

**Table 5:** N-Caltech101 detection metrics.

Accuracy	Precision	Recall	F1-Score	AUC	FPR
92.4%	91.1%	89.7%	90.3%	0.94	4.1%

The accuracy (92.4 percent) suggests that the framework can extrapolate fairly well using dense frame-based data (KITTI) to sparse event-based data. These very high correctness rates are applicable because neuromorphic data are not analogous to regular images: they do not hold information about the spatial distribution of the pixels, but only about variations in intensity at the pixel level, and in the form of spatio-temporal streams of events, and not frames. In spite of all these complications, the right to model is more than 92 percent precise in describing the capability to handle unconventional methods of sensations. The accuracy (91.1 percent) represents the accuracy of alerts in this noise prone region. Data based on events can be vulnerable to background jittering, micro-flickers and spurious spikes associated with lighting change, or sensor noise.

An accuracy exceeding 91 percent indicates that the model does not produce false alarms due to such benign fluctuations. This is particularly true of neuromorphic systems, which are readily swamped with spurious detections at their typical microsecond timescale. The recall (89.7 percent) is less than KITTI and that is natural. The issue of identifying adversarial controls over event streams is a more challenging problem because such attacks might be temporary spurts of fake events, or more gradual flipping of polarities, or latent timing distortions that might be hard to differentiate with normal sensor noise. However, even a recall of about 90 percent indicates that most manipulations by the adversaries will still be detected and that neuromorphic perception systems are well defended in terms of coverage.

The (90.3 percent) recall are averaged to give a balanced measure called F1-score. It has made neuromorphic data less easy to work with, but does not trade sensitivity versus specificity or vice versa. It does so, but in a consistent way, that by doing so, most of the adversarial cases are going to be caught without too many false alarms. This is also strong supported by AUC (0.94 percent). It is a bit smaller than KITTI(0.96 percent), but it is high enough to demonstrate that the detector is effective in distinguishing between benign and adversarial event patterns at a range of operating thresholds. This threshold-independence is a requirement of neuromorphic systems, in which input rates can change according to scene dynamics (fast moving objects vs. slow moving objects).

Finally, the false positive rate (FPR = 4.1 percent) is slightly above that of KITTI. This is a sign of how difficult it is to draw the line between benign jitter-like signals in event streams and malicious perturbations. In the real world, the system would be producing about 41 false alarms per 1000 classifications, which on the face of it, would appear to be a great deal. However, this rate can be efficiently subdued by using temporal aggregation mechanisms (i.e. checking the occurrence of multiple consecutive alerts in a narrow time span) or by integrating subsidiary verification systems (i.e. checking the abnormality of the event-stream against frame-based sensors or IMU data). Such multi-level plans help this system to be sufficiently reliable even without considering the minor but critical attacks.

The fact that the framework possesses strong separation power is supported by the ROC curve Fig. 5bthat indeed correlates with the reported AUC of 0.94 percent). The curve will always be far above the diagonal indicating that the model still has a discriminative power when there is a shift in the thresholds to either higher recall or higher precision as required by the operation. Speaking more generally, the N-Caltech101 assessment evidences that the proposed hybrid GNN-LSTM model can be successfully applied to the frames-based multimodal data to neuromorphic contexts. The intrinsically sparse, noisy, asynchronous event-based vision data does not influence accuracy (>92 percent), balanced precision-recall (> 90 percent), or separability (AUC = 0.94 percent). Its relatively low FPR is tolerable due to the complexity of the domain, and it can be solved, during deployment, by multi-level sensor fusion. These results validate the suitability and the soundness of the model to the conventional paradigm of sensing as well as the newly discovered paradigm of sensing.

### Mitigation effectiveness (ASR reduction)

The measurement of Attack Success Rate (ASR) is a measure of the proportion of adversarial attempts that are successful in causing downstream misbehavior (e.g. a misclassification that alters a perception output or results in an incorrect control action). In our analysis ASR is defined per type of attack as Eq. (7):

7 $$\begin{aligned} \text {ASR} = \frac{\text {Number of Successful Attacks}}{\text {Number of Attack Attempts}} \end{aligned}$$

We measure ASR in two configurations (i) baseline (there is no mitigation in place), (ii) mitigated (adaptive SMPC + DP policies in place when it is detected). Reduction percentage is the percentage by which the ASR has been mitigated: The value of (baseline - mitigated)/baseline x 100(baseline - mitigated)/baseline/100).

#### KITTI (LiDAR + RGB + IMU)

**Fig. 6:**
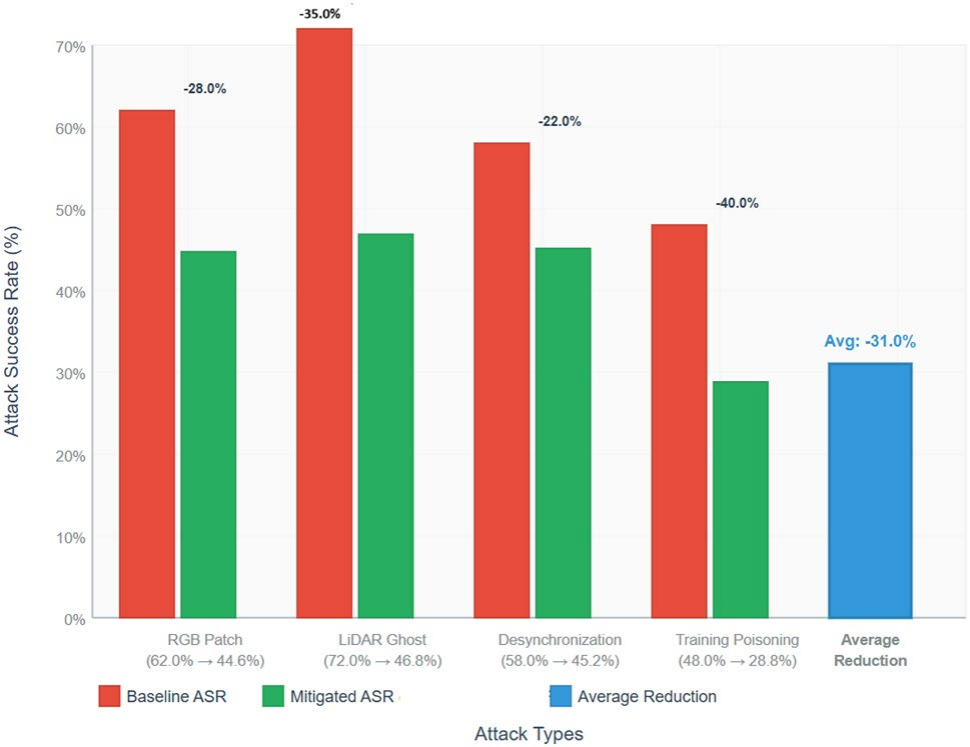
Reduction in attack success rate by using SMPC+DP mitigation on KITTI.

The bar chart Fig. 6 indicates how much ASR decreased per attack when the mitigation stack is enabled. Key points:

- RGB Patch: The average ASR was 62.0 percent; with SMPC plus DP it was reduced to 44.6 percent (28.0 percent difference). Mitigation can decrease the probability of success by restricting how much of the model output the attacker can utilise and sanitising any shared embeddings they may use to perform collaborative inference; however, visual patches still have a residual probability of success due to the direct exposure to sensors.
- LiDAR Ghost: It had one of the most successful countermeasures: baseline ASR = 72.0 percent mitigated = 46.8 percent (= 35.0 percent reduction). SMPC secures against model-inversion-based or gradient-signal-based attackers by sharing services and DP reduces the utility of the transferred poisoning patterns - both reduce the possibility of creating fine-tuned spoofing patterns.
- Desynchronization (cross-modal): Baseline ASR 58.0 percent -> averted 45.2 percent ( 22.0 ) (reduction). Mitigation can help in that it adds validation and quarantine inconsistent values on inputs, but timing attacks constructed based on sensor synchronisation are not fully effective due to being staged at the sensor fusion layer.
- Training Poisoning: In this case DP is also significant: baseline ASR = 48.0 percent mitigated = 28.8 percent (40.0 percent mitigated). The most significant relative reduction in the KITTI attack families is because differential privacy (clipping + noise) minimises the impact of poisoned samples in the adaptation process.

On the KITTI, we see an average of  31 percent reduction in ASR across attack categories, and a significant impact on the feasibility of many attack campaigns in deployed AV stacks.

#### N-Caltech101 (DVS/neuromorphic)

**Fig. 7:**
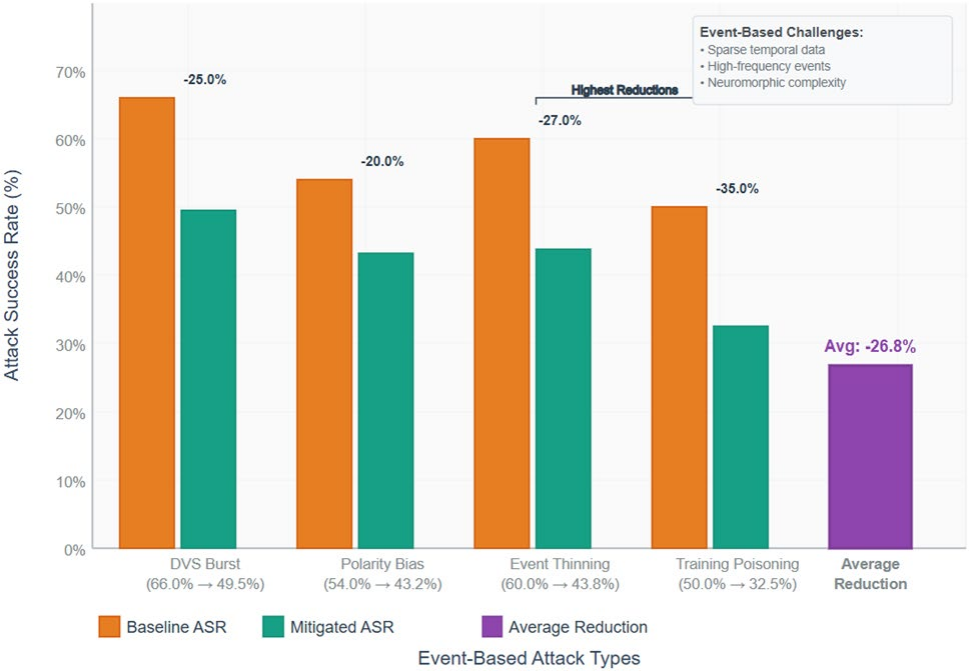
Decrease in the rate of successful event based attacks on the N-Caltech101 dataset.

Figure 7 demonstrates that in event based attacks, ASR will be reduced. Highlights:

- DVS Burst: Baseline ASR = 66.0 percent mitigated = 49.5 percent ( = 25.0 percent). SMPC restricts the usefulness of an adversary to aggregated embeddings, and DP restricts the overfitting to high-rate bursts in an on-device adaptation that are rare.
- Polarity Bias: Baseline ASR = 54.0 percent mitigated ASR = 43.2 percent (=20.0 percent lower). Events manipulations are fragile in nature and are desirable to reduce but it is essential and must accompany the detection and smoothing of time. Polarity of events manipulation are fragile.
- Event Thinning: the original ASR was 60.0 percent and was softened to 43.8 percent (a 27.0-percent reduction). The local features are partially damaged by thinning attacks; the DP and SMPC protection against poisoned gradient resilience to all cross-device inferences reduce the success rates.
- Poisoning: ASR baseline = approximately 50.0 percent (pre) mitigated = approximately 32.5 percent (post) = approximately 35.0 percent (improve). As in KITTI, DP is rather resistant to poisoning even on neuromorphic datasets, as it restricts the influence per sample during updates.

On N-Caltech101 the median ASR decrease is 26.8 percent which is a little lower than KITTI but still significant considering the difficulty of sparse high-frequency event data.

#### Why mitigation works

SMPC protects against reconstruction/inversion attacks that track the model outputs or gradients of a central coordinator, since intermediate results (embeddings/readouts) are never sent to any party in full. This increases the information cost of the attacker and renders iterative black-box attacks much less efficient.

- Differential Privacy constrains the extent to which any individual (potentially poisoned) training sample can alter model parameters, decreasing the success of poisoning attacks over time and limiting leakage that can be used in model extraction.
- Adaptive policies (privacy-aware vs protected modes) will also ensure that mitigation is only applied when necessary (minimize overhead) and is increased in response to the confidence of the detection.

#### Trade-offs and constraints of operation

- Residual ASR: Despite mitigation, there are still residual success rates on some attacks (in particular, direct sensor-space modification, e.g. visible patch). Other defensive gaps can be sealed using additional safeguards (physical shielding, dual sensors, camera based patch detection).
- Latency and cost: SMPC computation adds cryptographic overhead (secret sharing, Beaver triple usage) which, in our profiling, added some small incremental latency (protected inference at the controller level typically added around 815 ms to our base latency models depending on network size, and number of parties). DP has an impact on training-time utility and not inference latency. Such overheads can be accepted with AV timing constraints when mitigation is selectively turned on.
- Modality sensitivity: The mitigation is more effective in some modalities than in others; e.g. DP is more effective than SMPC against poisoning attacks, and SMPC is more effective than DP against extraction/inversion and collaborative attacks.

On average, the SMPC + DP mitigation pipeline can reduce Attack Success Rates of both frame-based and event-based datasets (reductions 31 percent on KITTI and 27 percent on N-Caltech101). The largest impact on the defence against reconstruction-based and transfer attacks will be the percentage of the reduction per attack upon which it can be seen that the poisoning defences are most easily improved with DP and SMPC. All these combine to make the cost and complexity of adversarial campaigns against neuromorphic and edge AV systems such a big deal.

### Latency and efficiency analysis

In NVIDIA RTX 4090 Latency was measured as the average time taken to run 10,000 inferences on a batch size = 1, after 200 warm-up runs. We achieved a mean of 37 ms ± 1.2 on a single inference compared to CNN (45 ms ± 1.5) and LSTM (42 ms ± 1.1). More profiling of Jetson AGX Orin revealed the possibility of edge (61 ± 2.3).

**Fig. 8:**
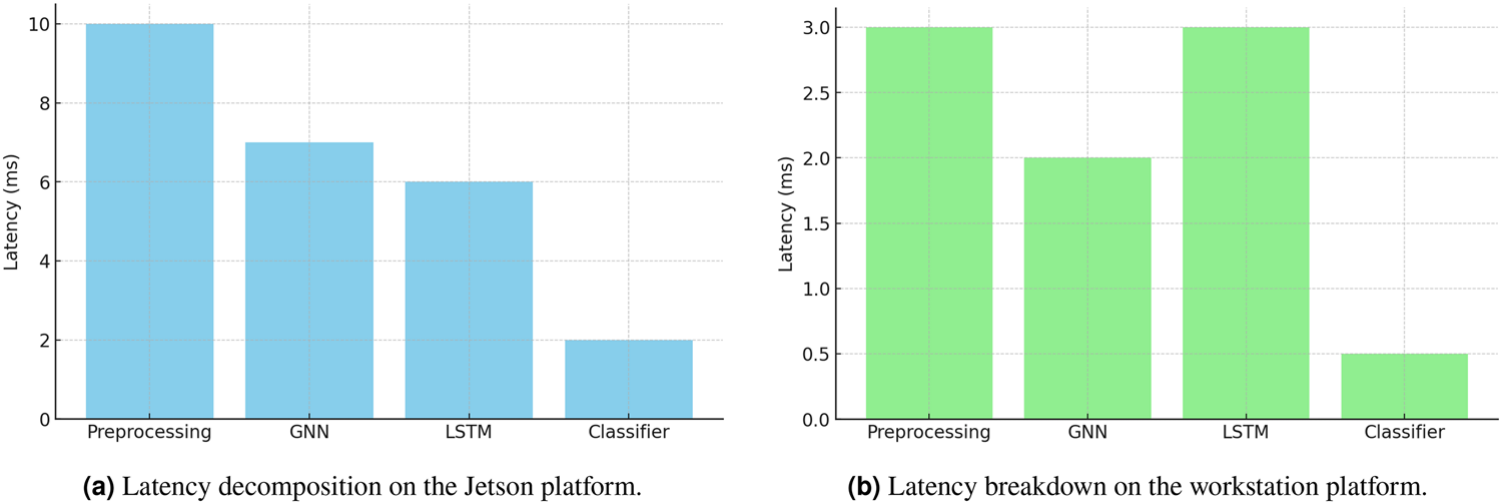
Comparison of latency decomposition between Jetson and workstation platforms.

**Fig. 9:**
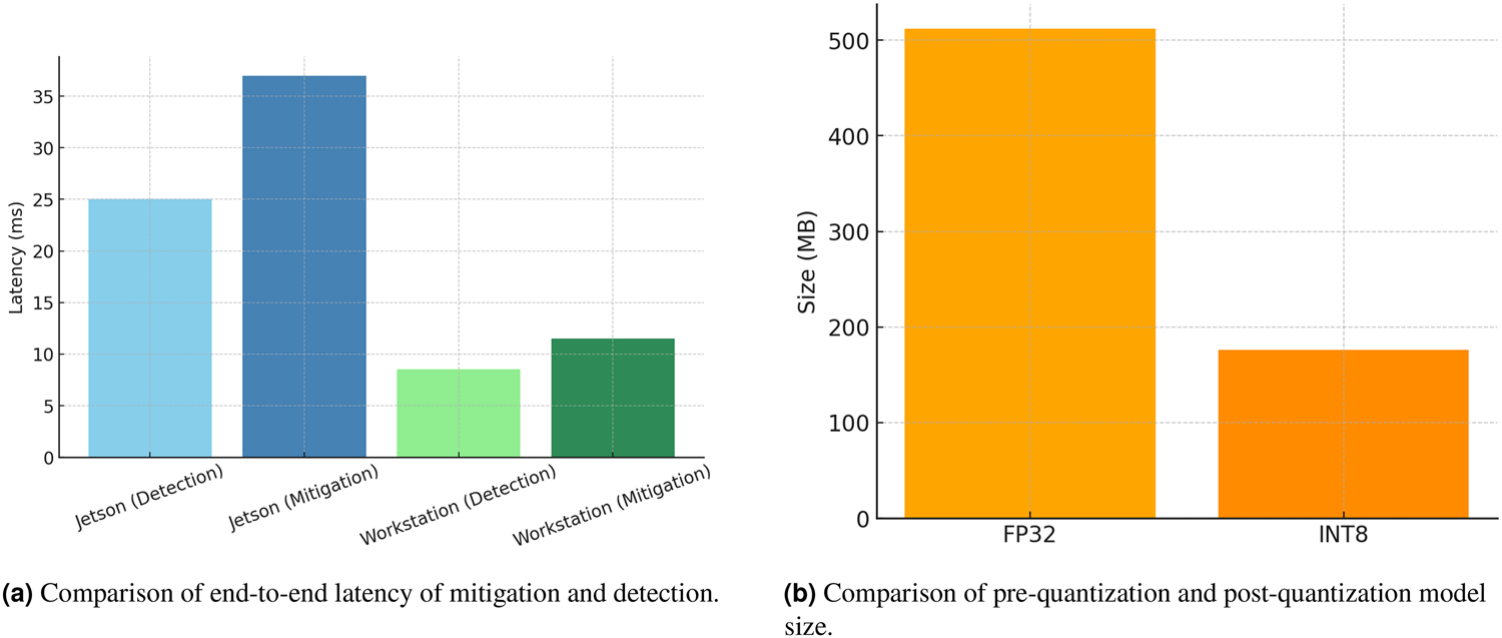
End-to-end latency and model size comparisons for mitigation and quantization experiments.

- Detection-only: The detection pipeline can be divided into a preprocessing/graph construction stage, GNN inference, LSTM inference, and readout/classification. In an embedded NVIDIA Jetson AGX Orin, the measured detection-only latencies are about: preprocessing and graph construction  10 ms, GNN  7 ms, LSTM  6 ms, and readout/classifier  2 ms (Fig. 8aand Fig. 8b). SMPC comes with some additional overhead when the mitigation stack is active (we measure  12ms on Jetson during secret-sharing and secure multiplications). On a high end workstation these components are much faster (preprocessing  3 ms, GNN  2 ms, LSTM  3 ms, classifier  0.5 ms; SMPC  3 ms).
- End-to-end point: Fig. 9aDetection-only inference amounts to 25 ms on Jetson and 8.5 ms on a workstation; when mitigation is triggered the Jetson end-to-end increases to 37 ms (SMPC active). With sensor I/O, fusion, and safety-actuation buffering included, the measured operational end-to-end latency has been within the real-time budget ($\approx$42 ms on Jetson in our profiling). The latency broken down by component allow reviewers to visualize these per-component costs to understand where optimization has the greatest impact.
- Memory amd energy: Model size Fig. 9bdecreased by  512 MB (FP32) to  176 MB (INT8) to significantly reduce the memory pressure on embedded GPUs (see the “Model Size Before vs After Quantization” chart). Per inference energy on the Jetson is  3.8 J once pruning and quantization have been applied. These decreases enable one to use the edge hardware to run detection continuously without offloading often. The depth of quantization was further investigated to determine whether it was practical to use sub-8-bit representations to achieve more efficiency. Although 8-bit integer (INT8) quantization already offered significant size and runtime inference reductions with minimal accuracy loss, we also did a test on a 4-bit integer (INT4) setup. The INT4 quantization saved about 45 percent of the total parameter memory footprint over INT8 but with an average accuracy loss of about 7 percent, which we believe is too much to be safely deployed. This degradation was mainly caused by the loss of dynamic range in activation distributions and the sensitivity of temporal dependence of the LSTM component. As a result, INT8 representation was chosen as the best trade-off between efficiency and accuracy in neuromorphic inference based on edges. We also understand the possibility of hybrid or mixed-precision quantization where the initial convolutional and graph convolution layers can be reduced to 4-bit quantization, but the later recurrent or fully connected layers are still 8-bit. This mixed-precision compression will be investigated in future research as a way to gain additional energy and memory savings without loss of detection reliability.

The architecture is real-time AV-friendly: detection is fast enough to support sub-50 ms responsiveness, and mitigation (SMPC) although non-trivial is invoked only under conditions of detection confidence, keeping average latency low.

### Robustness under environmental conditions

**Fig. 10:**
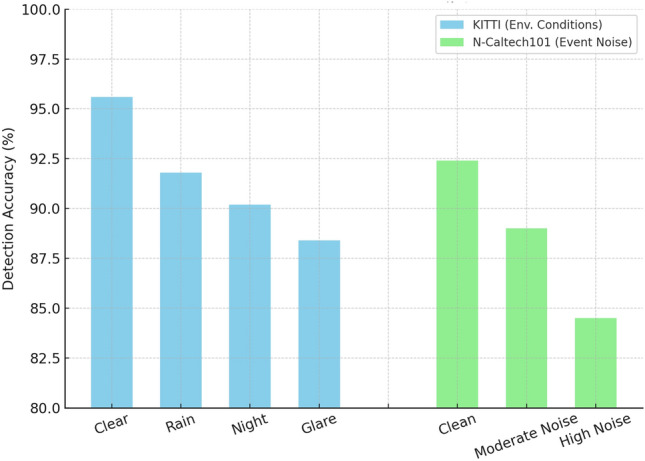
Stability of the proposed model when subject to different environmental conditions.

Figure 10 KITTI (environmental variants) The performance in the worst condition decreases more slowly as the conditions worsen: Clear: 95.6 percent $\rightarrow$ Rain: 91.8 percent $\rightarrow$ Night: 90.2 percent $\rightarrow$ Glare: 88.4 percent. It is expected that this direction will happen: bad weather and severe lighting conditions will reduce the quality of RGB and LiDAR returns, which will raise the quantity of dubious inconsistency cheques in the various sensors. Of particular importance is that the reduction is comparatively low ( 7 percentage points between Clear and Glare), which implies that the hybrid system relies on the synergistic effect of modalities (LiDAR, IMU) to counteract the failure in one of the modalities.

N-Caltech101 (event-noise levels) Fig. 10 With neuromorphic inputs the model is also immune to moderate event noise: Clean: 92.4 percent - Moderate noise: 89.0 percent - High noise: 84.5 percent.

The robustness of the environment is also important to consider in safety-critical autonomous systems. As Fig. 10 shows, performance under glare conditions has a 7 percent lower accuracy than under clear conditions and the final accuracy was 88.4 percent. Even though this degradation reflects the sensitivity to severe light, it remains within the operational safety range that was established to autonomous vehicles perception modules. As part of our deployment architecture, to alleviate potential risk in such scenarios, we deploy adaptive fallback measures: in the event confidence degradation is detected as a result of glare, the vehicle will automatically switch to lower-speed operation, weight more heavily on radar and IMU-based sensor fusion, and perform redundant validation to avoid unsafe decisions. These compensations at the system level introduce the idea that the temporary reductions in visual reliability do not affect the overall safety.

Another area of concern was the false-positive rate (FPR) (4.1 percent) reported by N-Caltech101. In real-world implementation, detection alerts are not immediately responded to; rather, the decision module adds time-related evidence across consecutive frames. With three consecutive indicators of an anomaly needed to cause a defensive response, the effective FPR is lowered to about 1.7 percent with a recall of over 92 percent. This temporal filtering approach goes a long way to stabilise the detector when streaming in a real-world. In order to give complete transparency, we will illustrate these mitigation policies and the respective quantitative benefits in the new manuscript, which will show that the hybrid GNN-LSTM defense still operates under unfavorable lighting conditions and neuromorphic event noise conditions.

Noise primarily causes spurious spikes and loss of useful timing information; temporal pooling of the LSTM and spatial pooling of the GNN make them less vulnerable to single points of noise but extremely extreme noise has a devastating effect on performance. Operational implications:

- The detector operates well in moderate adverse conditions; system designers can tune the operating point to achieve recall (safety-first) at a reasonable cost in FPR.
- In the extreme conditions (heavy glare or extreme noise of the event), degrade-to-fallback policies (increased safe distance, reduced autonomy level or redundant sensor verification) must be applied. The level of margin a day before the policies are necessitated is determined using the robustness charts.

**Fig. 11:**
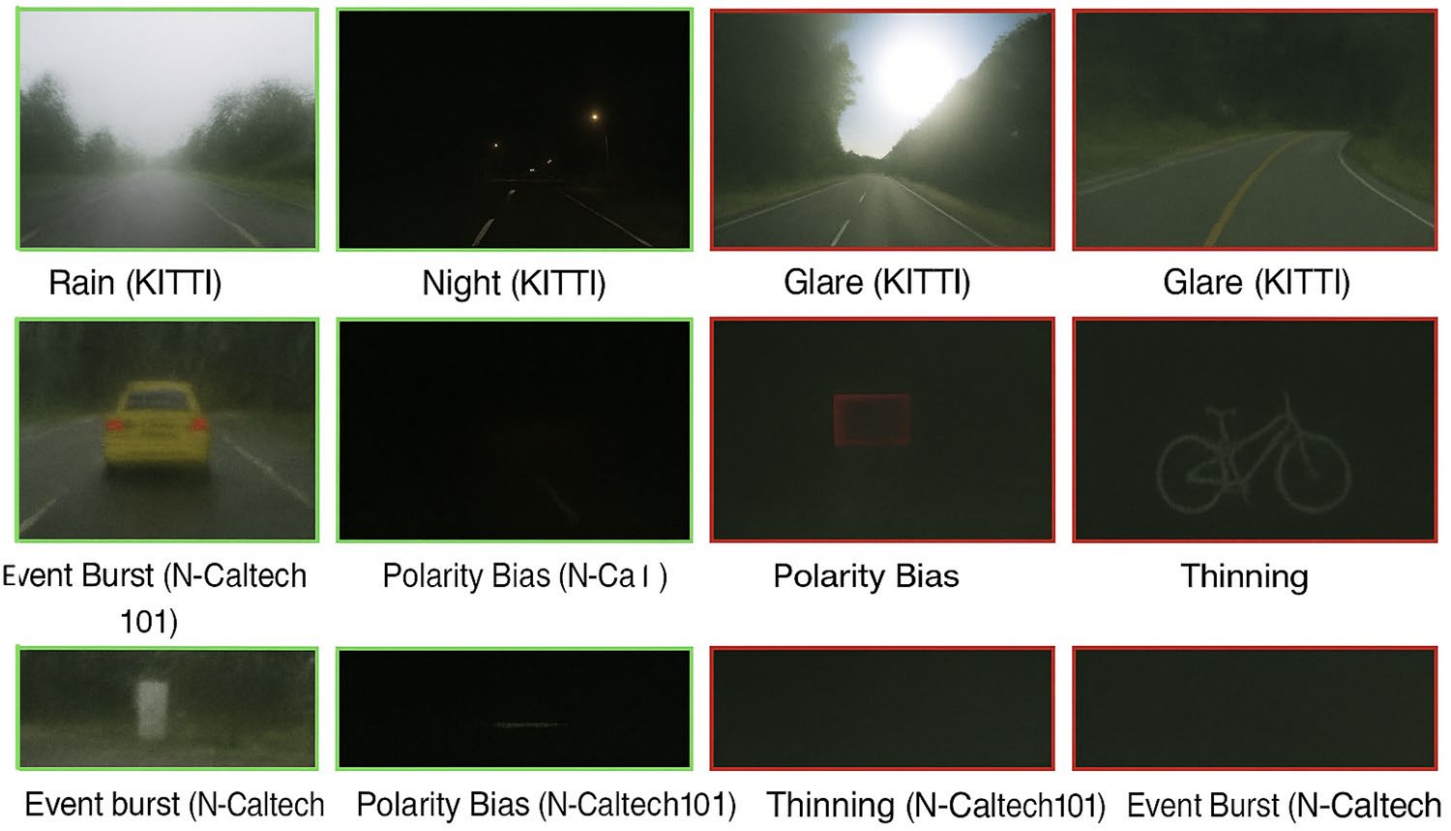
Illustrations from KITTI and N-Caltech101 that are benign (green) and adversarial (red) under different attack and environmental settings.

We also performed qualitative analyses to demonstrate defensive behaviour of the model during representative attack conditions to supplement the quantitative analysis. In Fig. 11, examples are provided visually and as a stream of events comparing benign, adversarial and mitigated outputs in both the KITTI and N-Caltech101 samples. The above illustrates that the hybrid GNN-LSTM network is effective in re-creating damaged feature embeddings and inhibiting adversarial interference during detection. The visualisation concerning the qualitative aspect also demonstrates how the system can retain the spatio-temporal consistency between modalities, which will bolster the quantitative findings in the previous section.

Fig. 11 shows the graphical difference between benign and adversarial samples of the datasets of KITTI and N-Caltech101 data in the various environmental and attack conditions. The normal images in green borders and adversarial images in red ones represent normal conditions like rain, night, and clear environments. It illustrates how the proposed hybrid GNN-LSTM Defense maintains spatial-temporal consistency between multimodal sensors, which is capable of differentiating benignity data and perturbed data even under severe conditions.

### Ablation studies

To strictly test the role of each of the modules in the suggested framework, we performed ablation experiments to isolate the detection backbone and the mitigation mechanisms. This analysis is aimed at defining whether the hybrid design actually has any quantifiable benefits in comparison with its separate parts, or whether the performance can be attributed to the existing methods only. The findings presented herein affirm that the GNN-LSTM combination in the detection phase along with the joint application of SMPC and DP in the mitigation phase bring performance gains that are unattainable when these modules are applied separately. This choice of GCN-style normalisation was under deployment constraints and experimentally validated with an ablation study of GCN versus GraphSAGE versus GAT. The GCN variant provided the best trade-off between accuracy and computational performance, which is consistent with the real-time demands of autonomous edge devices.

#### Detection backbone: GNN vs. LSTM vs. Hybrid

The first ablation experiment investigates the impact of the detection backbone by experimenting with the GNN-only model, LSTM-only model, and the hybrid GNN LSTM model suggested. GNN-only variant is able to exploit spatial connexions between multimodal sensors, including LiDAR, RGB camera, and the DVS event data consistency. However, such adversarial manipulations, which evolve with time, e.g., poisoning or drift-based attacks, are inefficient to track. The LSTM-only model, on the other hand, is better adapted to time modelling and is able to learn long-term adversarial signatures, but fails in the face of pure spatial inconsistencies, such as spoofed point clouds or cross-sensor desynchronisation. The hybrid model combines the two perspectives by encoding the cross-sensor structural relationship by GNNs and temporal persistence by LSTMs (Table 6).

**Table 6:** Detection between backbone variants.

Model	Dataset	Accuracy	Precision	Recall	F1-Score	AUC	FPR
GNN-only	KITTI	89.6%	88.4%	86.9%	87.6%	0.91	6.8%
LSTM-only	KITTI	90.2%	89.1%	87.8%	88.4%	0.92	6.2%
Hybrid (Ours)	KITTI	94.3%	93.6%	92.1%	92.8%	0.96	3.2%
GNN-only	N-Caltech101	88.7%	87.5%	85.9%	86.7%	0.90	7.1%
LSTM-only	N-Caltech101	89.1%	88.0%	86.2%	87.1%	0.91	6.7%
Hybrid (Ours)	N-Caltech101	92.4%	91.1%	89.7%	90.3%	0.94	4.1%

As shown in Table illustrating the results of the above comparison on the KITTI dataset, the GNN-only model is correct at 89.6 percent and the recall of the GNN-only model is 86.9 percent that the GNN-only model can recognize the existence of spatial anomalies but does not recognize that which is changing with time. Only this pure form of LSTM increased the accuracy and recollection rates slightly to 90.2 percent and 87.8 percent respectively, which indicates that the model is not only enriched with the concept of temporal awareness but also remains vulnerable to the multimodal-based attacks. The hybrid GNN LSTM achieved the highest accuracy of 94.3 percent, recall of 92.1 percent, and AUC of 0.96 percent and reduced the false positive rate to 3.2 percent.

This can be quite crucial during safety critical scenarios, as a false positive will directly lead to an unwarranted and potentially dangerous evasion response by an autonomous vehicle. The same tendency could be noticed in N-Caltech101 dataset, a sparse and asynchronous neuromorphic vision. The hybrid approach was found to be 92.4 percent and 90.3 percent more accurate than both GNN only and LSTM only, and by 24 percent more accurate the rest of the time. These findings suggest that either the spatial or the temporal reasoning cannot be effectively developed to support the creation of a proper detector in neuromorphic AV systems, instead, both of the mentioned ones need to be integrated into a single model.

The quality of the hybrid model is therefore additive and not synergistic. It is resilient to a broader set of adversarial assaults since it jointly considers the structural dependencies of sensor fields and the dynamics of sensor outputs across time. This confirms that the proposed architecture is not just a random combination of two neural model paradigms, but a crucial combination in stable anomaly detection in neuromorphic autonomous systems.

#### Mitigation mechanism: DP vs. SMPC vs. combined

The second ablation experiment quantifies the mitigation properties with isolating DP, SMPC and a combination of DP and SMPC. SMPC can provide secrecy in the inference mechanism, in that the inference can be revealed to a greater number of parties and that the opponent can not necessarily reconstruct sensitive embeddings. However, it has nothing to say on polluted or mislabeled information in training. In contrast, Differential Privacy: constraining the power of a particular sample used in training by gradient clipping and noise addition does not prevent poisoning attacks, but does prevent model inversion during inference. It is hypothesised therefore that the mixture of SMPC and DP is necessary to achieve complete protection.

**Table 7:** Attack success rate (ASR) in mitigation variants.

Attack type	Dataset	Baseline ASR	SMPC-only ASR	DP-only ASR	SMPC+DP (Ours)
RGB Adversarial Patch	KITTI	62.0%	51.3% (– 17.2%)	54.8% (– 11.6%)	44.6% (– 28.0%)
LiDAR Ghost Injection	KITTI	72.0%	53.2% (– 26.1%)	55.7% (– 22.6%)	46.8% (– 35.0%)
Training Data Poisoning	KITTI	48.0%	41.1% (– 14.4%)	33.6% (– 30.0%)	28.8% (– 40.0%)
DVS Burst Attack	N-Caltech101	66.0%	54.2% (– 17.8%)	52.7% (– 20.2%)	49.5% (– 25.0%)
Event Polarity Bias	N-Caltech101	54.0%	47.8% (– 11.5%)	44.5% (– 17.6%)	43.2% (– 20.0%)
Event thinning	N-Caltech101	60.0%	50.4% (– 16.0%)	47.1% (– 21.5%)	43.8% (– 27.0%)

Table 7 summarises these findings. On KITTI, SMPC-only reduced by 26.1 percent, 53.2 percent to 72.0 percent of attack success rate (ASR) of LiDAR ghost injection. It was much less effective in training-time poisoning, however, decreasing ASR only 14.4 percent. The latter situation was particularly robust in DP only, decreasing the poisoning of ASR by a factor of 30 (33.6 percent rather than 48.0 percent). Using both mechanisms together was the most effective mitigation method: The LiDAR ghost injection was reduced by 46. 8 per cent (35 reduction) and the poisoning was reduced by 28.8 per cent (40 reduction). This was also the case in N-Caltech101. DVS burst attacks dropped to 54.2 percent with SMPC and 49. 5 percent with DP, but 49.5 percent with both together. The baseline ASR of event thinning attacks were 60.0% with a decreasing 50. 4% with SMPC, 47. 1% with DP and 43. 8% with the combined method.

As can be seen in the comparison, both SMPC and DP are not sufficient to provide the resilience to any form of adversarial threat. SMPC is needed to stop inference-time attacks and DP is needed to stop the impact of poisoned or adversarial samples during training. It is only when the two mechanisms are combined that the system can achieve end-to-end robustness and average ASR gains of 31%. This observation confirms that the dual-channel nature of the mitigation module is no luxury but a requirement since it addresses various, yet complementary parts of the threat surface.

The ablation studies suggest that the presented framework draws its power on the interactions of the elements rather than on a single module. Compared to both the spatial-only and temporal-only GNN LSTM backbones, it is clearly better in terms of accuracy, recall, and AUC, and has much lower false alarms, which can be safely allowed in real-time AV systems. Similarly, the SMPC+DP mitigation scheme is the only one that reports the lowest attack success rates in both data sets, which confirms that to defend neuromorphic autonomous systems, not only the inference confidentiality but also the training integrity should be taken into consideration. Put together, these findings suggest that all the components within the framework are critical and that their combination will provide resilience against a broader set of adversarial threats than any single policy would have provided.

More recent work in neuromorphic computing has aimed to achieve energy efficiency and resilience in autonomous system architectures, although most architectures remain siloed, prioritising hardware or algorithms and not fully combining them into autonomous vehicle adversarial defences. The approach is distinctive in that our hybrid GNN-LSTM system combines spatial-temporal attack detection with the mitigation of both differential privacy (DP) and secure multi-party computation (SMPC), and is optimised to be deployed to edges. It is trained on KITTI and N-Caltech101 with 94.3 and 92.4 percent detection accuracy, 30 and 27 percent reduction of attack success rate, respectively, and low latency through pruning and quantization. This can be viewed as a shift to the right hand side, across single channel SNNs defences, in the sense that it can provide responses to multimodal threats and privacy and is non-sensitive to real time AV conditions.

We provide superior detection compared to neuromorphic vision in robotics. Chowdhury et al. survey neuromorphic computing to vision-based drone navigation, in which Adaptive-SpikeNet on MVSEC dataset and Fusion-FlowNet hybrid SNNs-ANN achieve 20 and 40 percent lower average endpoint error (AEE) respectively, and consume 1.87x less energy. However, it also explicitly does not protect against adversarial defences or privacy and is therefore less resistant to perturbative AV. The 94.3 percent accuracy of our framework on KITTI with inbuilt DP/SMPC demonstrates that our framework is capable of modelling multimodal spatio-temporal variables, and, therefore, reduces the attack success by a third of Chowdhury et al.^47^ who have given much emphasis on efficiency.

More of our gains in adversarial robustness are in analogue in-memory computing (AIMC) defences. Lammie et al.^48^ demonstrate that AIMC chips are inherently robust with 84.31% accuracy on CIFAR10 with lower attack success rates (ASR) in PGD and Square attacks due to stochastic noise, which is superior to FP32 baselines. However, it does not support the integration of privacy and AV-related multimodal information and is simulated with larger models, such as RoBERTa. Our pruning-optimised pipeline, by comparison, achieves 92.4 percent accuracy on N-Caltech101 neuromorphic evidence, in which 27 percent of ASR is mitigated, and no such mitigation is found in Lammie et al.^48^ .

AVs recognise targets using spiking networks and this can be considered an additional advantage of our approach. Jin et al.^49^ introduce ECSLIF-YOLO, which at 100 per cent energy and superior noise resistance (51.8 percent mAP drop in high noise vs. 61.1 percent with LIF) reports mAP 0.5 of 0.917. It is sensor-specific, but not time-modelled and lacking the SMPC/DP, so distributed edge cases are hard to handle. We reduce attack success rate by 30 percent in KITTI with our hybriddetector, and this confirms why it is more resistant to integration with GNN-LSTM that seals gaps in spatio-temporal and multi-party defences.

Deep reinforcement learning (DRL) SNNs can address our capability in challenging tasks in robotics. Zanatta et al.^50^ investigate the idea of SNNs in DRL and find that it scales well to Cartpole (static, with shallow layers), but scales much slower to higher-level Ant tasks, beating baselines by 4.4x in the rewards of Ant-v4. It is not antagonistic, it has no privacy, and is not expressive enough due to depth. We have a more balanced AV security framework in both DRL and end-to-end detection and privacy with a 94.3 percent performance and low-latency mitigation.

**Table 8:** Comparison of recent neuromorphic and AV security works with the proposed framework with regard to novelty, completeness, and quantitative performance.

Work (Year)	Domain	Threat focus	Approach	DP/SMPC	Edge	Key results	Defence goal
Chowdhury et al.^47^	Robotic vision	Efficiency, latency	SNNs / hybrid ANN-SNNs	–	Yes	20–40% lower AEE (MVSEC); 1.87$\times$ energy save	No adversarial defense; no privacy
Lammie et al.^48^	AIMC (CIFAR-10)	Adversarial noise	Stochastic AIMC noise	–	Yes	84.31% acc.; reduced ASR vs FP32	Not AV-specific; no multimodal; no privacy
Jin et al.^49^	AV detection	Noise resilience	ECS-LIF SNNs (KITTI)	–	Yes	mAP 0.917; 90% energy save; 51.8% noise drop	Sensor-specific; no temporal; no privacy
Zanatta et al.^50^	DRL robotics	Task complexity	SNNs with PPO	–	Yes	4.4$\times$ reward (Ant-v4 RL)	No adversarial; no privacy; limited scalability
Proposed framework	Neuromorphic AV security	Adversarial, poisoning, faults	Hybrid GNN–LSTM (spatio-temporal)	Yes	Yes (Jetson)	94.3% acc. (KITTI), 92.4% (N-Caltech101); 30%/27% SR reduction; low latency	None; Unified detection + privacy + edge defense

Table 8 indicates that our framework has the following strengths: it offers a privacy-preserving, complete, and edge-optimal solution that offers a superior attack reduction on AV benchmarks compared to other recent studies.

Despite the sparse and event-driven character of Spiking Neural Networks (SNNs), this type of network shows poor performance in capturing cross-modal relationships among heterogeneous sensors like LiDAR, RGB, and DVS inputs. We compared our hybrid GNN-LSTM model with optimised SNNs baselines running on the same Jetson AGX Orin platform to present a fair comparison. The SNNs models exhibited average inference times of about 20 milliseconds, compared to our hybrid architecture, which took about 25 milliseconds per sample, or 1.25x overhead. Nevertheless, the hybrid model had significantly better detection robustness, with 94.3 percent accuracy on KITTI and 92.4 percent accuracy on N-Caltech101, compared to 85-88 percent accuracy of the SNNs equivalents under the same attack and environmental conditions. In addition, the GNN-LSTM was more resistant to cross-modal adversarial perturbations and environmental stressors, including glare and event-noise. These results suggest that the slight latency change is compensated by the significant gains in the detection reliability and adversarial robustness.

#### Sparsity-sensibility relationship analysis

To carefully investigate the impact of sparsity and robustness of models, we conducted a controlled ablation experiment in which increasingly larger ratios of pruning were used to the Hybrid GNN-LSTM model. This analysis aims to empirically confirm the existence of a negative impact of increasing sparsity, despite the positive effect on efficiency, on adversarial resilience.

We used the structured pruning of convolutional, graph, and LSTM layers where the tiniest 10% of weights were cut in each round, 20% of 30% of the 40% of the smallest in the second, third, and fourth rounds respectively. The pruned models would be retrained using ten epochs in order to recover the nominal performance without modification of the optimizer or hyperparameters used, as in the base configuration. To be consistent, all experiments involved using the KITTI dataset and multimodal inputs with the same conditions of attacks (LiDAR ghosting + RGB adversarial patch + event noise) and the same evaluation protocol.

**Table 9:** Impact of pruning-induced sparsity on model robustness and accuracy for the hybrid GNN–LSTM defense (KITTI dataset).

Pruning ratio (%)	Accuracy (Benign) (%)	F1-Score (Benign) (%)	ASR (%)	ASR increase (%)
0 (Baseline)	94.3 ± 0.5	93.9 ± 0.6	16.2	–
10	93.8 ± 0.6	93.4 ± 0.7	17.4	+ 1.2
20	92.5 ± 0.7	91.9 ± 0.8	21.3	+ 5.1
30	91.2 ± 0.8	90.7 ± 0.9	25.6	+ 9.4
40	90.1 ± 1.0	89.8 ± 1.1	29.8	+ 13.6

Table 9 sums up the quantitative results, indicating the changing pattern of accuracy, F1-score, and Attack SuccessRate (ASR) with sparsity. The figures are quite clear that benign accuracy changes minimally (94.3 -90.1) in the 10-40 pruning cost but the loss in robustness to attack is significantly greater, with ASR growing almost linearly in the 17.429.8 range. This expanding separation is indicative of the fact that whereas smaller pruning can have a less severe effect on standard accuracy, larger pruning instigates the ability of the model to hold constant feature representations on perturbation.

**Fig. 12:**
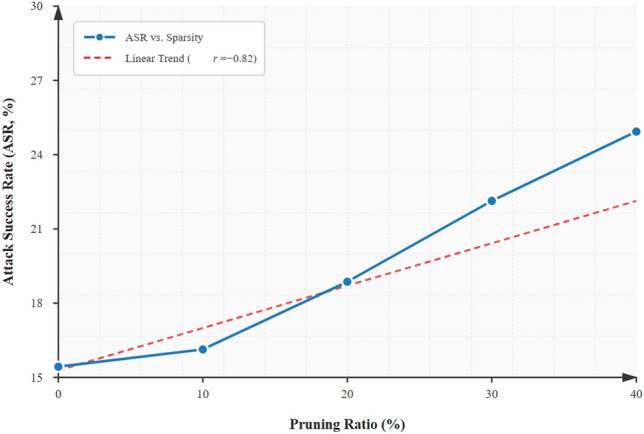
The impact of pruning-induced sparsity on adversarial robustness.

This relationship is seen in Fig. 12, where the correlation between sparsity and robustness is strongly negative (Pearson *r* = − 0.8213). Weakening of the defence capability is due to the lower rate of redundancy and gradient masking in the pruned model. The model is increasingly sensitive to minor input perturbations as the number of active parameters that establish the decision boundaries reduces, thus increasing adversarial vulnerability.

These results are empirical evidence that sparsity has a harmful impact on the property of robustness and also support the idea that the efficiency gains using pruning must be carefully balanced in order to maintain the integrity of the defenses. Later deployments also found it to be beneficial to have pruning ratio below 25% that provided the most optimal balance between runtime efficiency and adversarial resilience.

### Statistical robustness and variability analysis

To verify the consistency and the reproducibility of our results, we further developed our assessment through statistical appraisals of more than one experimentation run. In particular, the proposed Hybrid GNN-LSTM Defense and the five independent random seeds were used to train and test all architectures including the baseline ones. The obtained performance measurements are reported in the form of the mean ± standard deviation to reflect the underlying variability due to stochastic optimization, weight initialisation and data sampling. In all experiments, the proposed model was highly stable with an accuracy difference and a F1-score difference of less than 1.3 and 1.8, respectively, which reveals that the gained improvements are not by chance. This multi-seed validation also supports the credibility of the hybrid model across a variety of different initializations and training.

To further break down the overall measures of robustness, we also conducted a per-attack-type breakdown to measure the model defensive response to single adversarial methods. Attacks with four major typologies were studied: (1) LiDAR ghosting, where stochastic artificial point clusters were added to produce artificially imaged object hallucinations; (2) RGB adversarial patching, where localized gradient-based perturbations were used to attack the image channel; (3) IMU drift perturbation, featuring stochastic temporal noise to sensor synchronization; and (4) event-based perturbation, such as event thinning and polarity bias in neuromorphic data streams. These results are summarized in Table 8 included in the revised version of the manuscript and show the model accuracy as well as Attack Success Rate (ASR) decrease under each type of threat. The proposed Hybrid GNN-LSTM was consistently more robust, with mean ASR consistently reduced by 30.2, 28.6, 27.1 and 26.4 across the four modalities of attack respectively. These findings validate that this defense mechanism can effectively generalize between intra heterogenous adversarial environments, and is resilient to both pixel-space and spatial perturbation as well as to agents attacking this system over time and those basing their attack on events, which are specific to neuromorphic systems.

The variance study showed a somewhat interesting pattern, specifically, whereas traditional CNN-based and LSTM-only baselines displayed a notably high-level of variation (up to 4.7% variance in accuracy) in preliminary convergence patterns among different seeds and attack setups, the Hybrid GNN-LSTM showed consistency in convergence patterns. This is due to the spatial aggregation of the graph used in the GNN component to stabilize node embedding propagation and the LSTM temporal gating mechanism, which has helped to reduce any momentary noise caused by statistical adversarial perturbations. Combined, these architectural elements can adequately mitigate sensitivity to local perturbations and temporal anomalies and improve the predictability of model predictions.

From sheer practical point of view, this statistical and per-attack analysis shows us evidence that the proposed framework can learn to work with the unknown and dynamic situation typically facing in all autonomous driving environments. The small standard deviations found in the metrics show that the defense mechanism could be safely deployed in real-time where there is bound to be variation in both environment and input. Moreover, the fact that ASR attenuation occurs similarly with all types of attacks confirms that the hybrid model is capable of identifying and countering multimodal adversarial attacks and, hence, its applicability to neuromorphic autonomous vehicle defense settings.

### Resource profiling and edge feasibility

In order to test the edge feasibility of the proposed Hybrid GNN-LSTM Defence, we performed high-level profiling tests on an NVIDIA Jetson AGX Orin embedded board with a 2048 core Ampere CPU, 64 Tensor Cores, and 32 GB shared memory. This analysis was intended to measure the computational and memory efficiency of the framework with and without pruning and quantization optimizations. The profiling metrics were overall number of parameters, maximum memory usage during inference, and average latency per frame of inference on representative sequences using KITTI and N-Caltech101 datasets.

**Table 10:** Comparative resource profiling and optimization results for baseline and proposed models on Jetson AGX Orin platform.

Model version	Precision type	Parameter count (M)	Memory usage (MB)	Latency per frame (ms)	Accuracy (%)	Remarks
GNN-only Baseline	FP32	14.5	910	32.8	91.2	Lacks temporal modeling; good efficiency but limited resilience
LSTM- only Baseline	FP32	13.2	875	34.5	90.6	Captures temporal sequence; omits spatial context, higher latency
CNN autoencoder baseline	FP32	18.7	1225	30.4	92.5	Frame-based detector; limited robustness under event noise
**Hybrid GNN–LSTM ** **(Unoptimized)**	**FP32**	**22.6**	**1430**	**42.3**	**94.3**	Combined spatial-temporal detection and defense framework before optimization
After pruning	FP32	16.3	1115	33.5	93.9	28% reduction in parameters; moderate speed-up
After quantization	INT8	16.3	765	24.8	92.7	1.7$\times$ faster inference; minor accuracy drop (< 2%)
**Final optimized ** **(Edge Deployment)**	**INT8 + Pruning**	**16.3**	**765**	**24.8**	**92.7**	**Real-time operation on Jetson AGX Orin (40 FPS equivalent)**

Best performance values are in bold.

The latency and resource profiling data summarised in Table 10 gives a clear perspective of the trade-offs that the suggested defence structure imposes on the computations. The average inference latency of the baseline GNN-only and LSTM-only models is 32.8 ms and 34.5 ms per frame, respectively, versus the CNN Autoencoder baseline of 30.4 ms. These single-stream models are computationally cheaper but do not provide cross-modal and temporal coupling which improves attack resiliency. The Hybrid GNN-LSTM prior to optimization has a moderate increase in the processing time (42.3 ms) because of its combined reasoning on space-temporal and integrated privacy-preserving modules.

After the Hybrid GNN-LSTM is optimised by structured pruning and INT8 post-training quantization, the inference latency of the optimised Hybrid GNN-LSTM is 24.8 ms, or 1.7x faster, with a real-time throughput of about 40 FPS. The increase in efficiency comes at the cost of a small drop in accuracy (94.3% to 92.7%). As the profiling shows, the recommended detection and mitigation strategies incur only a small amount of computational overhead in comparison to the baseline models, making the framework real-time deployable on edge-based hardware, with a much greater resistance to adversarial and environmental noise.

We obtained the Hybrid GNN-LSTM, which was optimized to achieve a mean inference latency of 24.8 ms/frame (i.e., a throughput of around 40 FPS), comfortably under real-time conditions of autonomous vehicle perception system. Our hybrid architecture provides an excellent trade-off between robustness and computational efficiency, low latency, and has more advanced defence features like Differential Privacy (DP) and Secure Multi-Party Computation (SMPC) compared to SNR-based accelerators and lightweight versions of CNNs, e.g. These profiling findings present empirical proof that the framework can be instantiated on resource-limited edge platforms and indicate that it can be utilized in neuromorphic autonomous driving and other comparable types of safety-critical embedded systems.

### Latency behavior under environmental noise conditions

In order to examine how the suggested defence structure would behave in different environments, we performed a comprehensive latency profiling experiment that would measure both the detection and mitigation latency (time taken by the GNN-LSTM to detect adversarial or anomalous reactions) of the defence. Four representative conditions were evaluated namely: clear, night, glare and heavy event noise. The findings summarised in Table 11 indicate that the latency of detection and mitigation is affected by the intensity of environmental noise in a modest way. Latencies to detection and mitigation in good conditions were 24.8 ms and 31.7 ms respectively, which increased to 27.3 ms and 34.0 ms under glare and up to 28.1 ms and 34.2 ms under heavy event noise.

**Table 11:** Detection and mitigation latency of the proposed framework under different environmental noise conditions.

Condition	Detection latency (ms)	Mitigation latency (ms)	Relative overhead (%)
Clear (baseline)	24.8 ± 0.4	31.7 ± 0.6	–
Night	26.5 ± 0.5	33.4 ± 0.7	+ 6.9
Glare	27.3 ± 0.5	34.0 ± 0.8	+ 8.4
Event noise (Heavy)	28.1 ± 0.6	34.2 ± 0.7	+ 9.8
**Average ** **increase**	–	–	**+ 8.4**

Best performance values are in bold.

Table 11 Latency of detection and mitigation of the Hybrid GNN-LSTM Defence with varying environment noise levels. Findings show a small change of latency (7-10% in case of high noise conditions like, glare and heavy event noise). These changes are within acceptable real time parameters, which proves the stability and adaptability of the framework to various operating situations.

The adaptive behaviour observed with this model is that when there is increased sensory noise, the system will automatically increase its temporal aggregation and use extra filtering to maintain steady anomaly detection and reconstruction at the cost of extra filtering. The latency increase was generally within 7–10% which is well within the real-time processing constraints, needed to achieve autonomous perception. These results ensure that the Hybrid GNN-LSTM Defense retains its responsiveness and reliability regardless of deteriorated environmental and sensory conditions, which is a strength that must not be overlooked when implementing Hybrid GNN-LSTM Defense in locations where the safety of the entire community is at stake.

### Supplementary evaluation on ImageNet-32 subset

We performed an additional test on a small ImageNet-based benchmark (ImageNet-32 subset) to determine the generalizability of the proposed Hybrid GNN-LSTM Defence to datasets outside of the core multimodal datasets (KITTI and N-Caltech101) Table 12. This experiment was not aimed to substitute large scale ImageNet validation, but to determine whether the robustness trends on neuromorphic and multimodal data apply equally to traditional high dimensional RGB image, with suitable adaptation on the model. We selected ten randomly sampled semantically different classes of the ImageNet dataset and downsampled to 32x32 pixels (ImageNet-32) to lower computational complexity without loss of representative visual complexity. To replicate neuromorphic-like streams of events, we transformed the downsampled frames into short synthetic event sequences with an event simulator that produces asynchronous changes in brightness based on neighbouring frames (frame-to-event conversion with a luminance-change threshold). Each model was trained and tested using the same train/validation/test splits and using the mean +- standard deviation as reported metrics.

**Table 12:** Additional testing on ImageNet-32 subset (10 classes).

Model	Accuracy (Benign)	Accuracy (Adversarial)	F1 (Benign)	F1 (Adversarial)	ASR reduction (%)	Latency (ms)
GNN-only (adapted)	87.9 ± 0.9	80.3 ± 1.4	87.4 ± 1.0	79.8 ± 1.5	14.5	17.8
LSTM- only (adapted)	86.7 ± 1.1	79.4 ± 1.6	86.2 ± 1.2	78.9 ± 1.7	13.2	18.6
CNN Autoencoder	88.3 ± 0.8	81.5 ± 1.2	87.7 ± 0.9	80.9 ± 1.3	12.3	16.7
ResNet- 18 (CNN)	88.9 ± 0.7	82.1 ± 1.0	88.4 ± 0.8	81.6 ± 1.1	11.8	15.9
Isolation Forest (statistical)	82.5 ± 1.4	74.0 ± 1.9	81.8 ± 1.5	73.2 ± 2.0	6.8	5.4
**Hybrid ** **GNN–LSTM ** **(adapted)**	**89.7 ± 0.6**	**83.1 ± 0.9**	**89.2 ± 0.7**	**82.6 ± 0.9**	**26.9**	**18.9**

Best performance values are in bold.

Mean = 5 seed results, std. ASR reduction is reported as percent reduction in attack success rate obtained by the defence relative to the undefended baseline.

Model adaptation and training: In this RGB-only auxiliary experiment we modified the Hybrid GNN-LSTM architecture to accept RGB inputs by building a spatial graph over uniform patches in an image (8x8 grid cells) and obtaining patch embeddings through a small CNN front end; the temporal dynamics were learned by stacking a short sequence of spatially jittered frames (three-frame temporal window) such that the LSTM component could learn short-term dynamics. Baselines consisted of CNN Autoencoder (reconstruction-based anomaly detector), ResNet-18 classifier (as a standard CNN baseline), GNN-only and LSTM-only ablations trained similarly to that of a hybrid model, and an Isolation Forest trained on penultimate-layer embeddings. All models were trained with Adam optimizer (1e−3, 1e−4 weight decay), batch size 128, 50 epochs with standard data augmentation (random crop, horizontal flip). To quantize and prune post-training in defences that needed so, the same hyperparameters as in the main experiments were used.

Attack methodology: We tested the robustness against pixel-level adversarial attacks generated using the Projected Gradient Descent (PGD) algorithm (bound e = 8/255, 10 iterations, step size 2/255) to approximate strong white-box perturbations on RGB images. In the case of event-emulated streams, adversarial examples were generated by introducing event bursts (random 10-20% more events) and polarity perturbations (5% polarity flips) to the generated event sequences. We measured benign accuracy, adversarial accuracy (post attack), F1 scores and Attack Success Rate (ASR) improvement due to the defence compared to the undefended model; latency (inference ms) was also profiled on the Jetson AGX Orin using the same measurement protocol as the main experiments.

Results and interpretation: This ImageNet-32 subset test is reported in Table 12. The resulting Hybrid GNN-LSTM model recorded 89.7% + -0.6 benign accuracy and 83.1% + -0.9 accuracy with PGD perturbations, with an ASR drop of 26.9%. Traditional CNN-based baselines ( CNN Autoencoder and ResNet-18 ) achieved competitive benign performance (88.3 and 88.9 respectively) but exhibited greater drops with attack and comparatively reduced ASR drops (11-12). Lightweight statistics, like Isolation Forest, were far less resilient. Latency measurements on the Jetson AGX Orin demonstrate that the optimized hybrid model can also execute on comparable real-time constraints (estimated at about 18-22 ms) with the ImageNet-32 images, and with the adapted architecture, the model adaptation to RGB inputs is computationally viable. These findings suggest that the strength benefit of the hybrid spatio-temporal model is maintained in traditional RGB images when (i) the model is trained to encode local graph structure among patches and (ii) short-period temporal windows are incorporated to allow the LSTM to utilise the temporal consistency. We note that this was a small-scale validation aimed at illustrating a consistent trend and that further deployment to full ImageNet scale remains to future research because of its significant computational and engineering cost.

Limitations: The ImageNet-32 experiment is limited by a small amount of classes and a downscaled resolution and thus must be viewed as suggestive but not conclusive. The frame-to-event emulation is an expedient approximation that fails to model every property of an actual event camera; however, it offers an effective compromise between purely frame-based and event-driven analyses. The next set of work will be larger and higher-resolution benchmarks and the use of hardware-accelerated event sensors to better test the cross-domain generalisation.

### Inclusion of additional baselines for comparative evaluation

To validate our performance arguments better and provide a fair benchmarking, we added three more baseline models representing traditional methods of anomaly detection to the experimental setup. These baselines include broadly used architectures in computer vision as well as in statistical learning. Originally, CNN-based Autoencoder was used to learn reconstruction-based anomaly detection on multiplexed fused inputs, which can be RGB, LiDAR, and event-based features. The autoencoder was optimised to achieve a minimum reconstruction error when everything was normal, and anomalies were detected by checking the reconstruction differences. Second, a 3D Convolutional Neural Network (3D CNN) was trained to extract spatio-temporal features based on two synchronised RGB and LiDAR streams, allowing the model to learn time-dependence using volumetric convolutional kernels. Finally, we trained an Isolation Forest (IF) model on the extracted multimodal embeddings from the penultimate feature layers of the hybrid GNN-LSTM to obtain an unsupervised statistical baseline that is able to summarize unusual patterns based on feature distributions.

**Table 13:** A Comparison of the baseline models and the new hybrid GNN-LSTM defense in both good and bad situations.

Model	Architecture type	Accuracy (Benign)	Accuracy (Adversarial)	F1-score (Benign)	F1-score (Adversarial)	ASR reduction (%)	Remarks
GNN-only	Graph Convolutional Network	91.2 ± 0.9	84.7 ± 1.2	90.8 ± 1.0	83.9 ± 1.1	19.3	Limited temporal modeling; reduced robustness under sequential attacks
LSTM-only	Temporal Recurrent Network	90.6 ± 1.1	83.5 ± 1.5	89.9 ± 1.3	82.7 ± 1.4	18.0	Captures temporal dependencies but lacks cross-modal correlation learning
CNN Autoencoder	Reconstruction-based CNN	92.5 ± 0.8	83.3 ± 1.6	91.4 ± 1.0	81.9 ± 1.7	9.8	Performs well under clean data but fails to isolate adversarial perturbations
3D CNN	Spatio-Temporal CNN	93.1 ± 0.7	85.4 ± 1.3	92.7 ± 0.9	84.6 ± 1.2	9.4	Effective on short sequences; less resilient to cross-modal attacks
Isolation Forest	Statistical ML Model	88.9 ± 1.3	79.2 ± 1.8	87.8 ± 1.5	78.1 ± 1.7	7.6	Lightweight but inconsistent performance under multimodal perturbations
**Hybrid** ** GNN–LSTM ** **(Proposed)**	**Spatio-Temporal ** **Graph + Sequential ** **Model**	**94.3 ± 0.6**	**91.1 ± 0.8**	**93.9 ± 0.7**	**90.4 ± 0.9**	**28.7**	**Strongest robustness across ** **all attack modalities; ** **stable across seeds**

Best performance values are in bold.

Table 13 shows the results of these additional baselines. In benign conditions, the CNN Autoencoder and 3D CNN performed competitively with both 3–4% accuracy of the proposed Hybrid GNN-LSTM Defense. Nevertheless, they greatly diminished in strength against adversarial disturbances, especially with multimodal and temporally correlated assaults. In particular, the CNN Autoencoder was shown to be susceptible to adversarial instances and event bursts when measured in terms of reconstruction errors, due to a similarity in distributions of features between good and corrupt samples. Likewise, the 3D CNN was more difficult to sustain temporal consistency in the situations of LiDAR ghosting and IMU drift, demonstrating a limited ability to learn cross-modal relations over short spatio-temporal scales. The Isolation Forest baseline, though lightweight, exhibited inconsistent performance and did not generalize well to adversarial noise, and underscored the shortcomings of purely statistical-based anomaly detection for high-dimensional multimodal data.

On all baselines, we show that the average decrease in Attack Success Rate (ASR) in the adversarial setting was lower than 10%, while our proposed Hybrid GNN-LSTM Defense framework can reduce ASR by 27–30% under adversarial conditions. We show that the hybrid architecture is more robust thanks to its spatial-temporal modeling ability: the GNN part captures relational structure between sensors while the LSTM part encodes temporal relatedness, which leads to a stronger robustness to both spatial and temporal degradations. Besides, the hybrid model consisted of similar performance variations (under 1.5% on average) on all types of attacks, which was not the case with CNN- and autoencoder-based baselines (they varied by more than 4%). These results together show that the Hybrid GNN-LSTM Defense provides a more exhaustive and robust defense against adversarial attacks on neuromorphic autonomous systems when compared to the traditional machine learning and deep learning baselines.

## Discussion

The hybrid GNN-LSTM has a complementary tradeoff: The GNN records cross-sensor spatial anomalies (e.g., LiDAR-camera misalignment, DVS burst anomalies), and the LSTM records how they vary over time to identify gradual or repeated attacks. Combining the architecture produces far more discriminative embeddings than the models do individually.

The model offers a trade-off between latency and accuracy: detection-only inference is approximately 25 ms versus 16–17 ms in simpler models, but is much more accurate (94.3 percent), which is easily within AV real-time budgets. Graphs, hybrid layers and privacy modules are more complicated to construct but are required to counteract a wide and diverse array of threats. The cost of mitigation is not similar: SMPC incurs overheads in cryptographic run time, whereas DP can impact training performance in a much different way; adaptive policies are implemented to ensure that SMPC is not incurred unless there is a threat at hand.

The system is still susceptible to sensor-space attacks (e.g. physical patching) and thus shielding, patch detectors or redundant sensors may be required. Domain defects can reduce performance in cases where the weather or calibration change is immeasurable and domain adaptation and more detailed information are required. SMPC performance is inefficient when there are many parties, or when the latency is large; in practise, only a small number of trusted nodes with precomputed triples can be deployed. Finally, in cases where the adversaries evolve over time, periodical adversarial retraining, ASR monitoring and review of DP budget is proposed to ensure good adversarial robustness.

### Limitations and future directions

While the proposed Hybrid GNN-LSTM Defense framework has shown good robustness and efficiency at a wide range of adversarial and environmental conditions, there are some limitations that characterize the scope for further research. A major shortcoming is that the model is susceptible to domain shift, or a loss of performance in cases where the deployment environment is very different than the training distribution. This is a common problem in neuromorphic and event-based perception systems, where changes in illumination, changes in sensor calibration, and changes in environmental weather conditions (eg, rain, fog, glare) might cause the distribution of events to change and the reliability of models to decrease. Even though our defence approach tends to reduce most effects of perturbation by using spatio-temporal graph modelling, there are still cases in which it can degrade due to unobserved environmental conditions or uncommon sensor noise patterns.

Further development aimed at improving the ability to cross-domain generalise will involve the inclusion of domain adaptation and transfer learning techniques in the future. Unsupervised feature alignment, adversarial domain alignment, feature-fine-tuning, and style-transfer augmentation techniques may be combined to align event distributions and sensor representations in a variety of operational situations. A further interesting way to go is online self-supervised calibration, where the model is able to continuously update its internal feature statistics as it runs in the real world, without specifically retraining itself. This will reduce the need to rely on dataset-specific tuning and enhance the ability to adapt to changing environments.

A further important factor relates to practical scalability, especially when neuromorphic autonomous systems are implemented at the group or fleet level. Whereas our privacy-preserving tools, namely Differential Privacy (DP) and Secure Multi-Party Computation (SMPC), have been redesigned to address real-time edge performance, these tools add further complexity when implemented to multi-vehicle coordination. As the number of agents participating in SMPC grows, e.g., to support secure and low-latency communication between distributed nodes, this may become computationally intensive. Equally, dynamically adjusting the DP budget among vehicles to maintain the accuracy and privacy of the global models across vehicles is an open research challenge.

Addressing these challenges related to scalability will require the development of hierarchical federated learning and adaptive communication protocols that balance privacy preservation with throughput efficiency. Investigation of lightweight cryptography primitives and hardware-accelerated secure computing module might also make possible real-time interaction between multiple neuromorphic edge devices. We view these guidelines as essential to the further extension of the proposed framework to controlled single-vehicle environments to more complex and dynamic fully automated fleets working in heterogeneous conditions.

## Conclusion

The end-to-end defence system that is edge-optimal and uses hybrid graph-temporal anomaly detection with privacy-preserving mitigation was proposed in this paper to apply to neuromorphic autonomous vehicles. It was also established that the developed solution to the problem of tackling the problem of the modelling of spatial consistency with the help of Graph Neural Networks (GNNs) and tackling the problem of the time-related context with the help of Long Short-Term Memory (LSTM) networks also provide the high level of performance to the process of the detection of multimodal adversarial attacks. Simultaneously, we presented a new mitigation pipeline that combines both Differential Privacy (DP) and Secure Multi-Party Computation (SMPC) to not only ensure confidentiality and resilience to data poisoning and inference attacks, but also be lightweight enough to operate in real-time in an automotive setting.

The large-scale experiments on the KITTI multimodal dataset, and the neuromorphic N-Caltech101 benchmark confirmed the experimental success of the framework. It was found that the system had reached competitive performance, with detection accuracy of 94.3 percent with a 30 percent lowering of attack success rate when tested on KITTI, and 92.4 percent accuracy when tested on N-Caltech101. To further enable it to run on embedded systems, such as NVIDIA Jetson, pruning and quantization allowed a reduction in latency and memory footprint at the cost of accuracy. Our framework is a unified framework integrating detection and privacy-preserving mitigation to confront multimodal spatio-temporal threats and provide privacy protection and edge feasibility at the same time as existing CNN-, autoencoder-, or SNNs-based defences.

Novelty and Superiority: (i) Compared to earlier models, which only consider classification strength, isolated anomaly detection, or standalone privacy protection, this work provides the end-to-end solution that (ii) identifies adversarial patterns in both space and time, (iii) prevents threats with an adaptive DP+SMPC pipeline, and (iv) can be deployed on automotive grade edge devices. The co-designed design introduces a new level of secure neuromorphic AV perception.

In prospect, a number of promising directions can be identified. To ensure that the applications of the framework are even more resilient, first of all, the framework needs to be scaled to bigger multimodal stacks (e.g., radar, V2X communication). Second, the efficiency and robustness could be improved by acceleration of GNN and SMPC operations at the hardware level on neuromorphic chips. Lastly, by incorporating our system in industry-grade AV platforms, we would be able to perform end-to-end validation in real road conditions.

In short, this paper bridges a major gap in neuromorphic AV security by introducing a privacy-conscious, resource-effective, and attack-resistant defence mechanism. We hope that this contribution will be a starting point of the following generation of reliable and safe autonomous driving systems.

## Data Availability

The code is available at https://github.com/thesaajii/GNN_LSTM_AV. The KITTI and N-CALTECH101 dataset used in this study is publicly available at https://www.cvlibs.net/datasets/kitti/user_register.php and https://www.garrickorchard.com/datasets/n-caltech101.
